# Extreme resistance to *Potato virus Y* in potato carrying the *Ry*_*sto*_ gene is mediated by a TIR‐NLR immune receptor

**DOI:** 10.1111/pbi.13230

**Published:** 2019-09-04

**Authors:** Marta Grech‐Baran, Kamil Witek, Katarzyna Szajko, Agnieszka I. Witek, Karolina Morgiewicz, Iwona Wasilewicz‐Flis, Henryka Jakuczun, Waldemar Marczewski, Jonathan D. G. Jones, Jacek Hennig

**Affiliations:** ^1^ Institute of Biochemistry and Biophysics Polish Academy of Sciences Warsaw Poland; ^2^ The Sainsbury Laboratory University of East Anglia Norwich UK; ^3^ Plant Breeding and Acclimatization Institute‐National Research Institute Młochów Poland

**Keywords:** potato, PVY, extreme resistance, RenSeq, *Ry*_*sto*_, TIR‐NLR immune receptor

## Abstract

*Potato virus Y* (PVY) is a major potato (*Solanum tuberosum* L.) pathogen that causes severe annual crop losses worth billions of dollars worldwide. PVY is transmitted by aphids, and successful control of virus transmission requires the extensive use of environmentally damaging insecticides to reduce vector populations. *Ry*_*sto*_, from the wild relative *S. stoloniferum*, confers extreme resistance (ER) to PVY and related viruses and is a valuable trait that is widely employed in potato resistance breeding programmes. *Ry*_*sto*_ was previously mapped to a region of potato chromosome XII, but the specific gene has not been identified to date. In this study, we isolated *Ry*_*sto*_ using resistance gene enrichment sequencing (RenSeq) and PacBio SMRT (Pacific Biosciences single‐molecule real‐time sequencing). *Ry*_*sto*_ was found to encode a nucleotide‐binding leucine‐rich repeat (NLR) protein with an N‐terminal TIR domain and was sufficient for PVY perception and ER in transgenic potato plants. *Ry*_*sto*_‐dependent extreme resistance was temperature‐independent and requires EDS1 and NRG1 proteins. *Ry*_*sto*_ may prove valuable for creating PVY‐resistant cultivars of potato and other *Solanaceae* crops.

## Introduction

*Potato virus Y* (PVY), a member of the *Potyvirus* genus (Potyviridae family), is the most economically important virus of potato crops, affecting tuber yield and quality and resulting in losses of 10–90% depending on the year, cultivar and location (De Bokx and der van Want, 1987; Valkonen, 2007). PVY can also infect other *Solanaceae* species such as bell pepper, tomato and tobacco (Aramburu *et al*., 2006). PVY is mechanically transmitted by more than 40 aphid species in a non‐persistent manner (Brunt, 2001). Intracellular pathogens such as viruses are difficult to control chemically, and PVY outbreaks are managed by the destruction of infected plants or by pesticide treatment to reduce insect vector populations (Kopp and Bánfalvi, 2015). Selective breeding of plants with resistance to viral pathogens remains the best method to limit the impact of viral infections. PVY variants are highly variable at the biological, serological and molecular levels (Scholthof *et al*., 2011) and can be classified into different strain groups according to the host response. Virus strains PVY^N^ and PVY^N^W produce barely noticeable foliar symptoms, whereas PVY^0^ and PVY^C^ usually induce mild symptoms of infection such as leaf mosaic lesions, crinkling, leaf drop and dwarfing. By contrast, as well as inducing severe foliar symptoms, PVY^NTN^ infection leads to potato tuber necrotic ringspot disease, which severely affects tuber marketability (Schubert *et al*., 2007).

Plant defence responses to viruses are multifaceted and depend on several factors, including plant age and tissue type. For PVY, resistance genes provide the most effective and durable control. Two main types of resistance against PVY are present in potato: extreme resistance (ER), conferred by the *Ry* genes, and hypersensitive resistance involving programmed cell death (HR), conferred by the *Ny* genes (Valkonen, 2015). However, HR is not effective in restricting PVY in plants under some conditions (Vidal *et al*., 2002). By contrast, ER genes are broad‐spectrum and confer strong and durable resistance, characterized by lack of visible symptoms after inoculation (Flis *et al*., 2005).

Resistance genes against PVY infection were previously introduced into potato cultivars from wild or domesticated *Solanum* species. Ten genes for resistance to PVY and one gene for resistance to PVA (*Potato Virus* A, a potyvirus related to PVY) were mapped to four potato genome segments on chromosomes IV, IX, XI and XII (Valkonen *et al*., 2017; Van Eck *et al*., 2017). Some of these genes, for example *Ry*_*sto*_ and *Ry‐f*_*sto*_ from *S. stoloniferum*, were introduced into multiple potato cultivars worldwide and shown to confer durable resistance against multiple PVY strains. However, no genes conferring effective HR‐ or ER‐type response have been cloned and characterized to date (Valkonen *et al*., 2017).

Previous estimates suggested that controlling major diseases through the informed deployment of resistance genes could increase crop yields by more than 30% while reducing the application of chemical pesticides (Gebhardt and Valkonen, 2001). This might be achieved by using new breeding tools to select resistant genotypes that can then be deployed in new crop varieties (Armstrong *et al*., 2019).

Most plant resistance genes encode intracellular nucleotide‐binding leucine‐rich repeat (NLR) receptors. Some of these genes carry an N‐terminal TIR domain and are termed TIR‐NLRs, whereas others carry an N‐terminal coiled coil domain and are termed CC‐NLRs (Jones *et al*., 2016). Pacific Biosciences single‐molecule real‐time sequencing combined with resistance gene enrichment (SMRT RenSeq) (Witek *et al*., 2016) is a useful tool for the identification and assembly of full‐length NLR‐encoding genes that cosegregate with resistance phenotypes. SMRT RenSeq methodology is more robust and cost‐effective for monitoring NLR sequences than whole‐genome sequencing. All the currently known NLRs effective against viruses, nematodes and the late blight pathogen *Phytophthora infestans* can be tracked with SMRT RenSeq in potato, and their polymorphisms have been defined (Armstrong *et al*., 2019).

Demand is high for strong, durable resistance genes to combat PVY infection, and *R* genes conferring ER to PVY fulfil these requirements. The mechanisms underlying the ER response to viruses have not been fully elucidated, and identifying the network of factors contributing to effective pathogen resistance is of key importance.

The aim of this study was to use SMRT RenSeq to isolate the *Ry*_*sto*_ gene that governs the ER trait. Multiple TIR‐NLR‐encoding paralogues were identified that cosegregated with resistance. Paralogue *Ry*_*sto*_ conferred resistance to PVY and the related potyvirus PVA in different *Solanaceae* backgrounds through recognition of PVY and PVA coat proteins (CPs) as *avr* factors for *Ry*_*sto*_‐triggered immunity. Finally, investigation of the functional relationship between ER and HR showed that ER was EDS1‐ and NRG1‐dependent but was not dependent on salicylic acid (SA) level or temperature.

## Results

### *Ry*_*sto*_ originates from *S. stoloniferum*

ER to PVY was introgressed into commercial potato cultivars from three different wild *Solanum* spp. In European cultivars, resistance originates mostly from *S. stoloniferum* (*Ry*_*sto*_ and *Ry‐f*_*sto*_) and has not yet been overcome (Valkonen *et al*., 2017). Therefore, we set out to clone *Ry*_*sto*_ using dihaploid clone dH Alicja, which has PVY‐resistant *S. stoloniferum* in its ancestry. A diploid mapping population consisting of 391 F1 individuals was generated and evaluated for resistance to PVY by mechanical and graft inoculations as described by Flis *et al*. (2005). The segregation ratio of resistant versus susceptible progeny in the mapping population deviated from the 1:1 ratio expected for the segregation of a single dominant gene and was distorted towards resistance (149 susceptible and 242 PVY‐resistant F1 individuals).

### RenSeq combined with SMRT sequencing and bulked segregant analysis led to successful cloning of *Ry*_*sto*_

**Figure 1:**
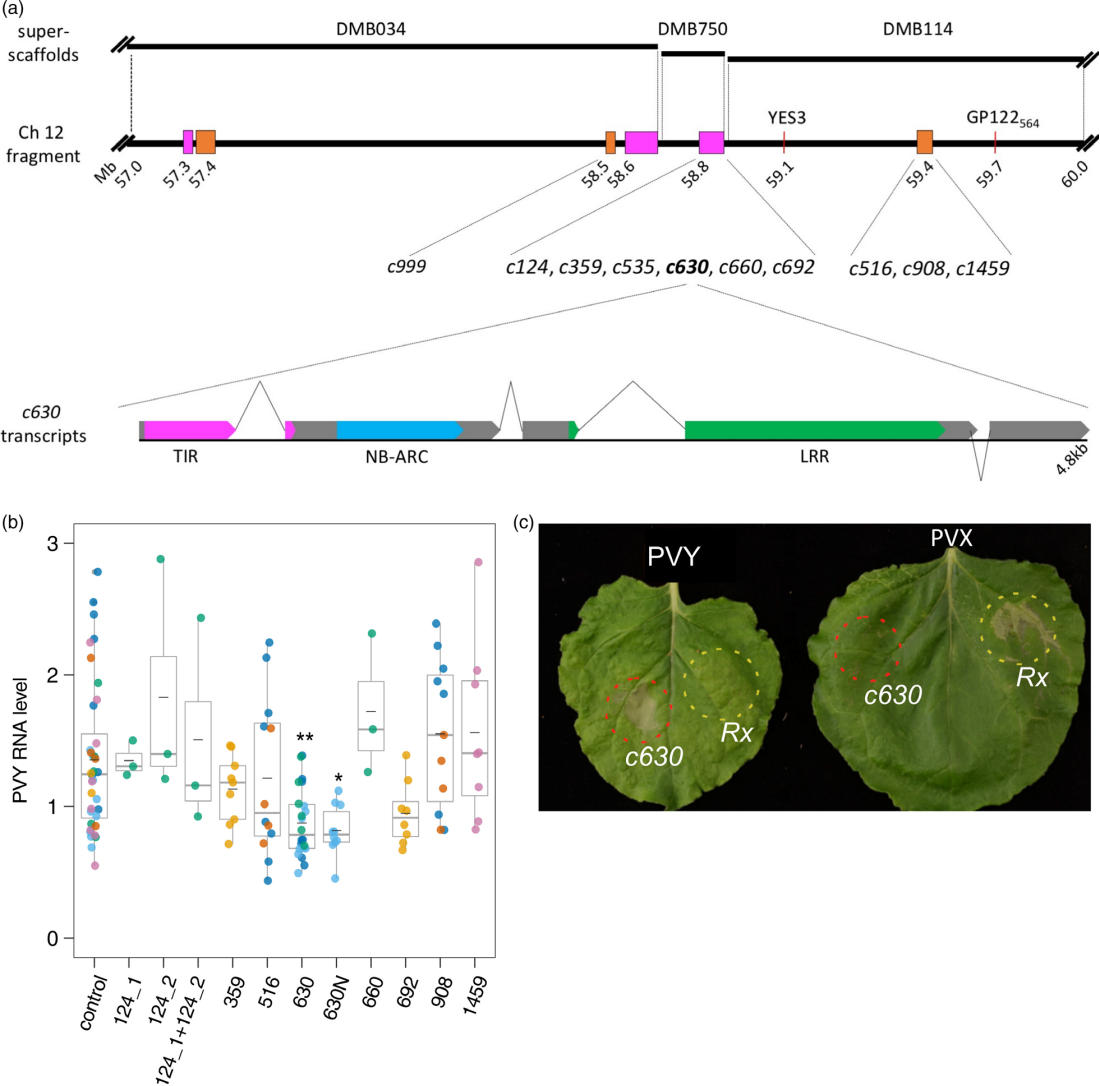
Functional analysis of candidate genes using transient expression assays in *Nicotiana benthamiana*. (a) Schematic representation of candidate genes and a fragment of chromosome XII linked to the *Ry*_*sto*_ gene. Potato superscaffolds (DMB034, DMB750 and DMB114; top panel) were aligned to the distal end of the longer arm of potato chromosome XII (57–60 Mb fragment shown; middle panel), which is linked to the *Ry*_*sto*_ gene. Annotated NLRs/NLR clusters are depicted in orange (CNL) and magenta (TNL). Red lines indicate known markers linked to *Ry*_*sto*_. Distances are given in Mb according to the DM potato reference genome (Potato Genome Sequencing Consortium (95 authors), 2011), with numbers indicating proximal positions of marked sequences. Candidate contigs for *Ry*_*sto*_ derived from SMRT RenSeq were aligned to the DM potato genome, and best hit positions are shown on the map. Map drawn to scale. Schematic structure of *c630* transcripts (lower panel). Approximately 80% of cDNA‐RenSeq reads supported a version of *c630* with four exons. An additional intron (depicted with inverted lines) positioned 24 nt upstream of the initial stop codon results in a fifth exon at the 3′ end. Exons are drawn in grey; colours indicate canonical NLR domains: TIR (magenta), NB‐ARC (blue) and LRR (green). Schematic drawn to scale. (b) Relative levels of PVY RNA after infection of *Nicotiana benthamiana* leaves transiently expressing candidate contigs. Three *N. benthamiana* leaves were infiltrated with vector pICSLUS0003:35S overexpressing *c124_1*,* c124_2*,* c359*,* c516*,* c630*,* c660*,* c692*,* c908 c1459* and pICSLUS0001 vector overexpressing *c630* under the control of native regulatory elements or an empty vector. After 48 h, leaves were inoculated with PVY^NTN^. Seven days after PVY inoculation, mRNA was isolated from upper, non‐inoculated leaves and PVY RNA levels were quantified with qPCR. *EF1* and *L23* were used as standardization references. Error bars represent the standard deviation of the means, and medians are also presented. One‐way ANOVA with Dunnett's test was used for statistical analysis. Experiments were performed on 3–10 plants for each construct and were repeated three times. (c) HR response of *N. benthamiana* plants expressing *c630* or *Rx* genes. Two fully developed leaves of *N. benthamiana* plants were infiltrated with a suspension of *A. tumefaciens* carrying pICSLUS0003:*c630* (area marked with red) or pGBT‐Rx‐GFP (area marked with yellow) 14 days after PVY^NTN^ (left) or PVX (right) infection. Seventy‐two hours after infiltration, expression of *c630* in PVY‐infected plants resulted in HR symptoms similar to those observed when *Rx* was delivered into plants infected with PVX virus. Experiments were repeated three times.

Previous research mapped *Ry*_*sto*_ to the distal end of the long arm of potato chromosome XII (Flis *et al*., 2005; Song *et al*., 2005; Van Eck *et al*., 2017), corresponding to the region downstream of 58 Mb in the DM reference potato genome (Potato Genome Sequencing Consortium (95 authors), 2011). Jupe *et al*. (2013) showed that this region contained sequences encoding 18 complete or partial NLR immune receptors from both the TIR‐NLR and CC‐NLR classes, organized in four clusters (Figure 1a). We therefore hypothesized that *Ry*_*sto*_ likely encoded a NLR and used RenSeq to predict candidate genes. SMRT RenSeq was used to assemble the NLR‐encoding genes of the resistant parent with high confidence. Assembly of Pacific Biosciences (PacBio) reads of interest (ROIs) resulted in 1555 contigs, 1254 of which were annotated as NLRs using NLR‐parser software (Steuernagel *et al*., 2006). Mapping of short‐read RenSeq data from resistant (R), susceptible (S) and bulked susceptible (BS) plants, and subsequent polymorphism calling, resulted in 10 linked contigs with presence/absence polymorphisms. Expression of candidate genes was confirmed using cDNA‐RenSeq data obtained from the resistant parent. Eleven candidate NLRs were identified from these analyses, four of which belonged to the CC‐NLR class and seven of which belonged to the TIR‐NLR class. A phylogenetic tree constructed with full‐length predicted amino acid sequences showed that the candidate genes belonged to three different clades and were only distantly related to cloned functional *Solanaceae* genes. All TIR‐NLRs belonged to the same clade and exhibited amino acid identities of 75–90%. The CC‐NLRs split into two distinct clades, consistent with their physical separation in the potato genome (Figure 1 and Figure S1).

Homology searches of the reference potato genome positioned the candidate genes in three clusters located at the distal end of the long arm of chromosome XII (58–59 Mb of the DM reference genome, Figure 1a), consistent with marker locations linked to *Ry*_*sto*_ (GP122_564_, Witek *et al*., 2006, and YES3, Nie *et al*., 2016). This region of chromosome XII consists of three neighbouring superscaffolds, DMB034, DMB750 and DMB114, one of which (DMB114) was proposed as a likely location for *Ry*_*sto*_ (Van Eck *et al*., 2017). All the selected candidate NLR genes from SMRT RenSeq data therefore localized to the distal end of chromosome XII in a region previously identified with *Ry*_*sto*_.

### Functional analysis of candidate contigs by transient expression in *Nicotiana benthamiana*

To determine whether candidate genes were functional *in planta*, the open reading frames (ORFs) of 11 cosegregating NLRs were PCR‐amplified and placed under the control of a *35S* promoter in the pICSLUS0003 binary vector, as described previously (Witek *et al*., 2016).

Constructs were transiently expressed in *N. benthamiana*, followed by PVY^NTN^ inoculation. At 7 dpi, leaf samples were collected and levels of viral RNA were measured using qPCR. One candidate gene (*c630*) reduced virus multiplication as compared to control *N. benthamiana* plants (Figure 1b). The *c630* ORF with its native 5′ and 3′ regulatory sequences (2263 nt upstream from 5′ and 1013 nt downstream from 3′ end, respectively) was cloned into the pICSLUS0001 binary vector as described (Witek *et al*., 2016). Transient assays in *N. benthamiana* also showed a statistically significant (*P *= 0.011475) inhibition of virus multiplication (Figure 1b). Candidate gene *c999* showed autoactivity, even when infiltrated with low OD (0.1), and another candidate gene, *c535,* could not be cloned.

Next, *N. benthamiana* plants were systemically infected with PVY^NTN^ or *Potato virus X* (PVX) prior to transient expression of *c630*. A HR response was observed upon expression of *c630* in plants carrying PVY^NTN^ (Figure 1c), similar to the response seen when the *Rx* gene was delivered into plants infected with PVX (Bendahmane *et al*., 1999).

### Expression of *c630* triggers HR in response to a range of viruses

To test whether *c630* elicited an immune response against different strains of PVY, *N. benthamiana* plants were infected with the PVY^0^, PVY^N^, PVY^N‐Wi^ and PVY^NTN^ isolates or cross related *Potato Virus A* (PVA). Leaves showing symptoms of viral infection at 7 dpi were infiltrated with *Agrobacterium* strains carrying *c630* or an empty vector. Transient expression of *c630* resulted in a strong HR in plants infected with all tested PVY isolates and related PVA, but not *Potato virus X* (PVX) or *Tobacco Mosaic Virus* (TMV) (Table S1). No HR was observed in control plants without *c630* expression. These experiments provided further support that *c630* was the functional *Ry*_*sto*_ gene.

### *Ry*_*sto*_ expression restricts systemic spread of PVY and PVA

To further investigate *c630*/*Ry*_*sto*_ function, stable transgenic *Solanaceae* plants (potato and tobacco) carrying *Ry*_*sto*_ or two other non‐functional homologues under the control of either a *35S* promoter or native regulatory elements were created using *Agrobacterium‐*mediated transformation.

**Figure 2:**
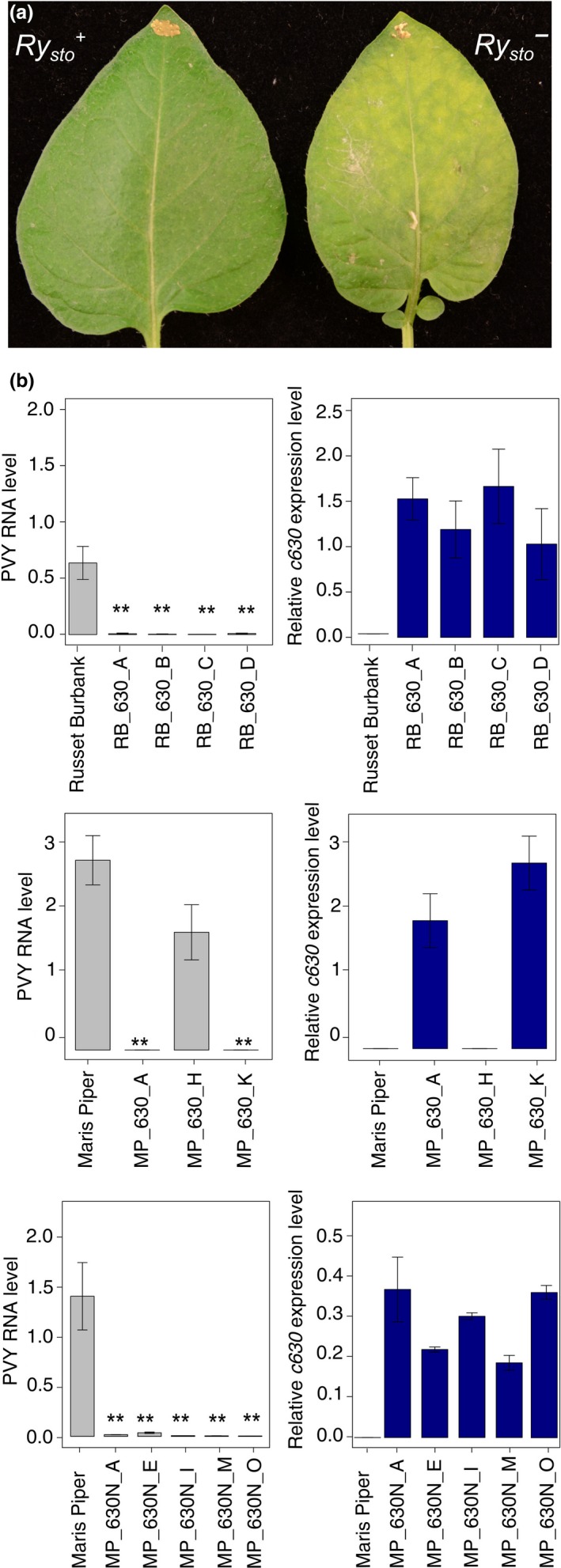
Expression of *Ry*_*sto*_ leads to immunity of transgenic plants to PVY infection. (a) Illustration of ER ‐type response of PVY ‐inoculated leaves of *Solanum tuberosum* cv Russet Burbank plants expressing *Ry*_*sto*_. Four‐week‐old transgenic potato plants cv. Russet Burbank carrying construct *35S:Ry*_*sto*_, and non‐transformed plants, were inoculated with PVY^NTN^ . Two weeks after infection, chlorosis was observed on inoculated leaves of non‐transformed plants (right), whereas *Ry*_*sto*_ transgenic plants (left) remained symptomless. (b) *Ry*_*sto*_ expression completely blocks PVY spreading in transgenic potato plants. Four‐week‐old transgenic potato plants cv. Russet Burbank (upper graph) or Maris Piper (middle graph) carrying construct *35S:Ry*_*sto*_, Maris Piper (lower graph) plants carrying *Ry*_*sto*_ construct under the control of native 5′ and 3′ regulatory elements and non‐transformed plants were inoculated with PVY^NTN^ . Three weeks after viral inoculation, mRNA was isolated from upper, non‐inoculated leaves. PVYRNA levels and expression of *Ry*_*sto*_ were quantified using qPCR , relative to *EF* *1* and *Sec3* reference genes, and expressed as means ± SD calculated from three biological replicates per plant line. One‐way ANOVA with Dunnett′s test was used for statistical analysis.

Two PVY‐susceptible potato cultivars *(*Maris Piper [MP] and Russet Burbank [RB]) were then used to test *Ry*_*sto*_ functionality. Transgenic plants of both cultivars carrying *Ry*_*sto*_ under the control of either a *35S* promoter or a native promoter (MP only) were inoculated with PVY^NTN^ or mock treated with water. Systemic virus spread was monitored in upper, non‐inoculated leaves using enzyme‐linked immunosorbent assay (ELISA) 3 weeks after inoculation. In the MP background expressing *Ry*_*sto*_ under control of *35S*, no PVY was detected in upper plant parts in 10 of the 12 tested transgenic lines (Table S2). To confirm these ELISA results, three randomly chosen transgenic lines (two resistant and one susceptible) were tested using qPCR (Figure 2b). The inhibition of virus multiplication and spread in the two resistant transgenic plants correlated with elevated levels of *Ry*_*sto*_ gene expression. In the susceptible transgenic line, no expression of the *Ry*_*sto*_ transgene was detected. No PVY was detected in RB in any of the four independently isolated transgenic lines, either by ELISA or by qPCR (Figure 2c and Table S3). Furthermore, all five lines in which *Ry*_*sto*_ was expressed under the control of its native promoter were PVY‐free (Figure 2d). No macroscopic symptoms of HR were observed in all resistant lines regardless of the genotype of potato used (Figure 2a).

To corroborate PVY resistance of MP, T1 generation progeny tubers of selected lines were collected and subsequently planted following by graft inoculation with tobacco scions carrying PVY^NTN^. In agreement with previous results, any symptoms of PVY infection were detected using ELISA assay (Figure S2; Table S4).

*N. tabacum 35S:Ry*_*sto*_ transgenic and non‐transformed control plants were inoculated with PVY^NTN^ as previously. At 5 dpi, some necrotic lesions were visible on the inoculated leaves of *Ry*_*sto*_ plants, whereas no macroscopic symptoms of HR were observed on the non‐transformed control plants (Figure S3). In addition, control susceptible plants developed typical systemic symptoms of PVY infection. qPCR analysis of upper, non‐inoculated leaves showed that systemic spread of the virus was fully restricted in *Ry*_*sto*_‐expressing lines, whereas PVY RNA levels in non‐transformed plants were high. These results were confirmed in T0 (Figure S4a) and T1 (Figure S4b) generation plants. Transgenic tobacco lines expressing *Ry*_*sto*_ under the control of its natural 5′ and 3′ regulatory elements exhibited a local HR response at 5 dpi similar to that seen in *35S::Ry*_*sto*_ plants. No viral RNA was detected with qPCR (Figure S5) at 7 and 14 dpi, and this corresponded with *Ry*_*sto*_ transcript expression.

Introduction of *Ry* genes from *S. stoloniferum* to *S. tuberosum* may condition resistance not only to PVY but also to PVA, which is closely related (Cockerham, 1970). To test whether expression of *Ry*_*sto*_ provided resistance to PVA, tobacco plants with and without the functional *Ry*_*sto*_ transgene were inoculated with PVA. Western blotting analysis with anti‐PVA antibodies revealed inhibition of PVA systemic spread in *Ry*_*sto*_ transgenic plants but not in control plants (Figure S6).

To compare *Ry*_*sto*_ and *Ry‐f*_*sto*_ alleles, a relevant region from PW363 breeding line was amplified using primers corresponding to the genomic sequence of *Ry*_*sto*_ (see Experimental Procedures). An alignment of these two *Ry* alleles revealed 100% sequence identity in the coding and non‐coding regions of the 4.85 kb amplified product (Figure S7).

### PVY and PVA coat proteins are elicitors of *Ry*_*sto*_‐triggered immunity

**Figure 3:**
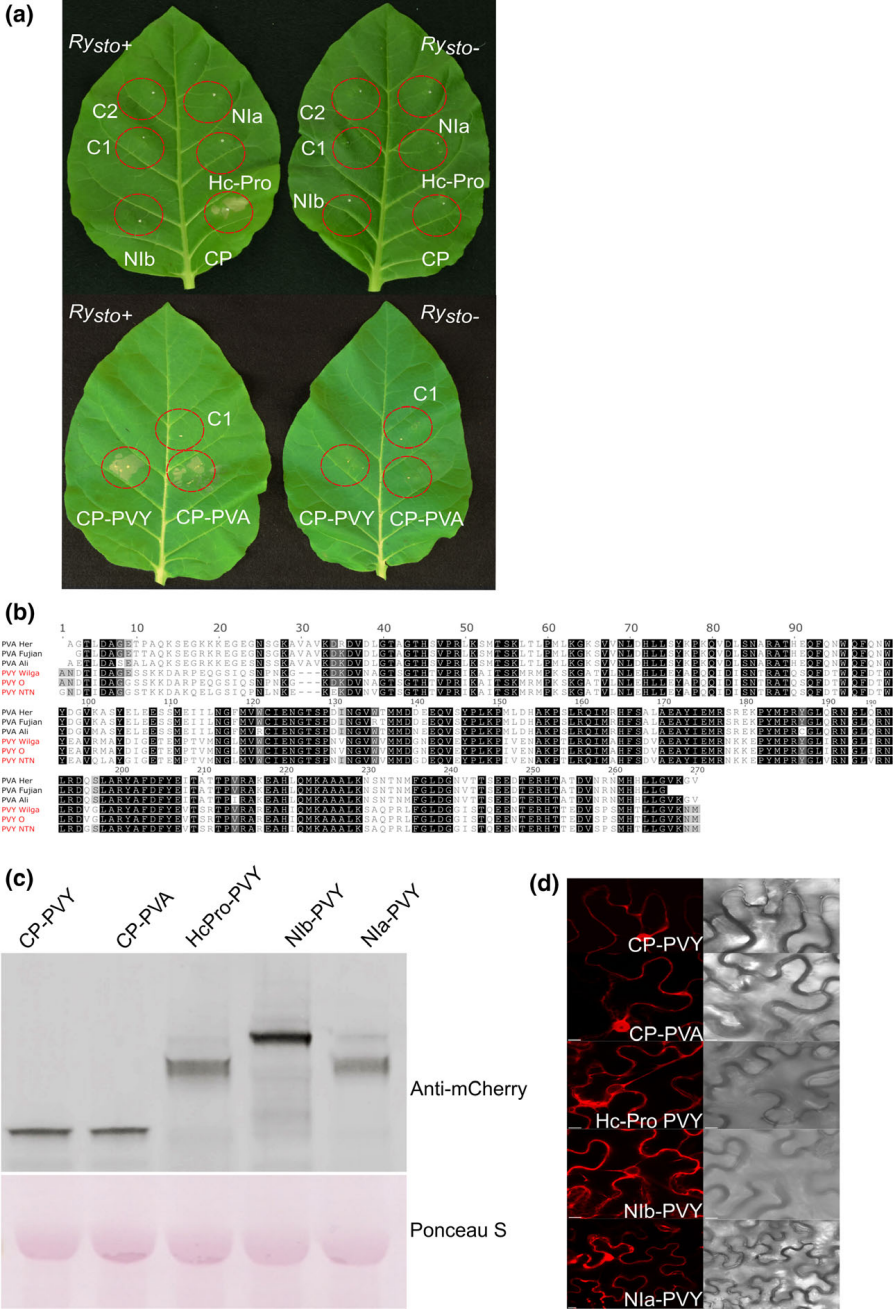
Coat proteins of two closely related potyviruses elicit *Ry*_*sto*_‐mediated immunity. (a) Ry_sto_ recognizes PVY (upper panel) and PVA (lower panel) coat proteins as avirulence factors in transient expression assays. To identify the elicitor of the *Ry*_*sto*_‐mediated resistance against PVY or PVA viruses, open reading frames encoding putative viral proteins (Hc‐Pro, Nia, Nib and CP) were cloned into the pBIN‐mCherry vector and transiently expressed in *N. tabacum Ry*_*sto*_ transgenic and non‐transformed plants. As a control (C1, C2), *Agrobacterium* with empty vector or buffer infiltration was used. Three days after treatment, HR was observed only for PVY or PVA coat proteins (CPs). (b) Comparison of PVY and PVA coat protein amino acid sequences. PVY and PVA CP amino acid sequences were compared with PVY^NTN^ CP. Identical residues are shaded in black. PVA CP shares >59% identity with the PVY sequence. The alignment was generated using MAFFT‐L‐INS‐I (Katoh and Toh, 2008) and visualized in Jalview 2,10,4b1 (Waterhouse *et al*., 2009). (c) Western blot analysis of viral protein expression. The indicated combinations of PVY‐CP‐mCherry, PVA‐CP‐mCherry, PVY‐Hc‐Pro‐mCherry, PVY‐NIb‐mCherry and PVY‐NIa‐mCherry were transiently expressed in leaves of *N. tabacum*. Total proteins were extracted and analysed by protein gel blotting with polyclonal anti‐mCherry antibody (Abcam). Staining of RuBisCO with Ponceau S was used as a loading control. (d) Cellular localization of viral proteins. Confocal images show representative *N. benthamiana* leaf epidermal cells transiently expressing indicated proteins. Images were taken 72 h after Agro‐infiltration. For each variant, approximately 50 transformed cells were examined. Bars = 10 μm.

A single ORF in the PVY genome encodes a polyprotein that is then cleaved by three viral proteases to form functional proteins (Jakab *et al*., 1997). To identify the elicitor of the *Ry*_*sto*_‐mediated immune response, the ORFs encoding putative viral proteins Hc‐Pro, NIa, NIb and CP were cloned and transiently expressed in transgenic *Ry*_*sto*_ and non‐transformed tobacco plants. Only the CP induced strong HR at 2–3 dpi in *Ry*_*sto*_ plants, whereas no HR was observed in control plants (Figure 3a). Similarly, only the CP of PVA elicited HR when expressed in transgenic *Ry*_*sto*_ plants (Figure 3b). The expression of specific viral proteins was detected by immunoblotting (Figure 3c), indicating that the absence of HR was not caused by a lack of protein expression. Additionally, cellular localization of each protein was determined (Figure 3d).

### ER mediated by *Ry*_*sto*_ is epistatic to *Ny‐1*‐mediated HR

**Figure 4:**
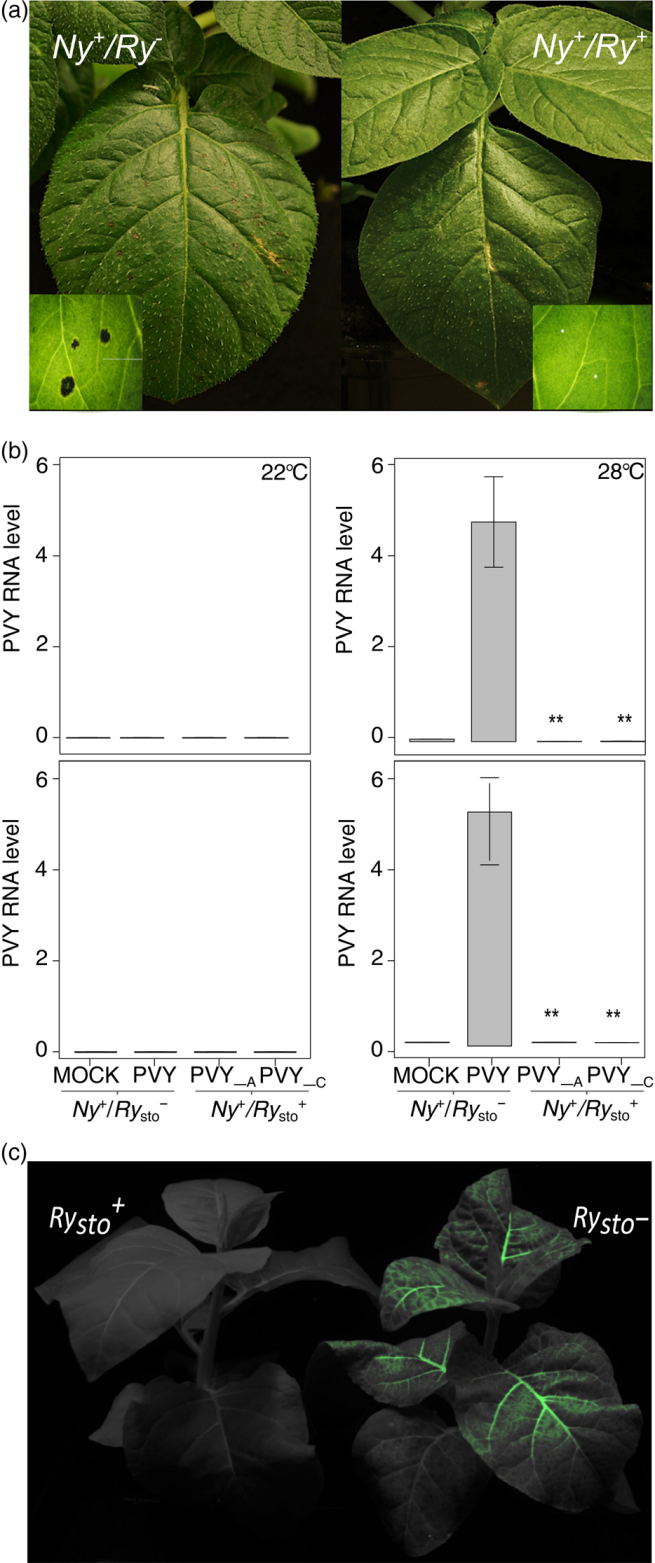
*Ry*_*sto*_‐dependent ER is epistatic to HR and is temperature‐independent. (a) Determination of epistatic effect of ER to HR. Potato *Ry*_*sto*_/*Ny‐1* and *Ny‐1* plants were inoculated with PVY^NTN^. Five days after infection, symptoms of local cell death occurred only in *Ny‐1* plants. The experiment was repeated three times. (b) *Ry*_*sto*_‐dependent PVY resistance is not inhibited at elevated temperature. Potato *Ry*_*sto*_/*Ny‐1* and Ny‐1 plants were inoculated with PVY^NTN^ and divided into two groups, at 20 °C and 28 °C. Water‐treated plants were used as negative controls. Seven days after inoculation, samples of inoculated (lower graphs) and upper non‐inoculated leaves (upper graphs) were collected and PVY RNA levels were measured using qPCR. Values are expressed relative to *EF1* and *Sec3* reference genes and are expressed as means ± SD calculated from three biological replicates per plant line. A and C describe the names of transgenic *Ry*_*sto*_ lines used. One‐way ANOVA with Tukey's test for statistical analysis was performed. (c) Stable transgenic *N. tabacum* plants carrying *Ry*_*sto*_ under the control of a 35S promoter are resistant to PVY infection at elevated (32 °C) temperature. Seven‐week‐old *N. tabacum 35S:Ry*_*sto*_ transgenic and non‐transformed plants were inoculated with a PVY^N^ ^205^:GFP clone. Typical symptoms of PVY infection were observed 7 dpi in non‐transformed plants, whereas *Ry*_*sto*_ lines remained symptomless. Image was taken at 14 dpi.

Next, the functional relationship between the two types of defence response to PVY infection, ER and HR, was investigated. Potato cultivar Rywal, carrying the *Ny‐1* gene responsible for HR to PVY infection (Baebler *et al*., 2014; Szajko *et al*., 2008), was transformed with *Ry*_*sto*_. Plants of both genotypes (*Ny‐1* or *Ry*_*sto*_/*Ny‐1*) were inoculated with PVY^NTN^, as described previously. Five days after infection, HR developed only in the parental genotype (*Ny‐1*), whereas no HR was observed in the *Ry*_*sto*_/*Ny‐1* genotype (Figure 4b). This indicated that ER conferred by *Ry*_*sto*_ was epistatic to *Ny‐1*‐dependent HR.

### ER mediated by *Ry*_*sto*_ is temperature‐independent

To investigate the role of temperature in *Ry*_*sto*_‐mediated ER, the response to PVY infection was studied in a *Ny‐1* or *Ry*_*sto*_/*Ny‐1* genotype. PVY mRNA levels were measured 14 dpi in upper non‐inoculated leaves following mock and virus treatments at two temperatures (22 °C and 28 °C). In both genotypes, no PVY multiplication was observed when plants were kept at 22 °C. By contrast, at 28 °C, HR was inhibited in the *Ny‐1* genotype and PVY RNA was detected systemically, whereas PVY spread remained fully inhibited in transgenic *Ry*_*sto*_/*Ny‐1* lines (Figure 4a and b). To test whether *Ry*_*sto*_ hindered systemic infection above 28 °C, transgenic and non‐transformed tobacco plants were infected with GFP‐tagged PVY (PVY^N605^‐GFP) at 32 °C. Expression of *Ry*_*sto*_ at the higher 32 °C temperature still prevented systemic spread of the virus (Figure 4c).

These observations suggest that high (>28 °C) temperature does not compromise *Ry*_*sto*_ immunity to PVY infection, unlike the tobacco N gene, which also encodes a TIR‐NLR (Samuel, 1931).

### NRG1 and EDS1 mediate *Ry*_*sto*_‐dependent HR

**Figure 5:**
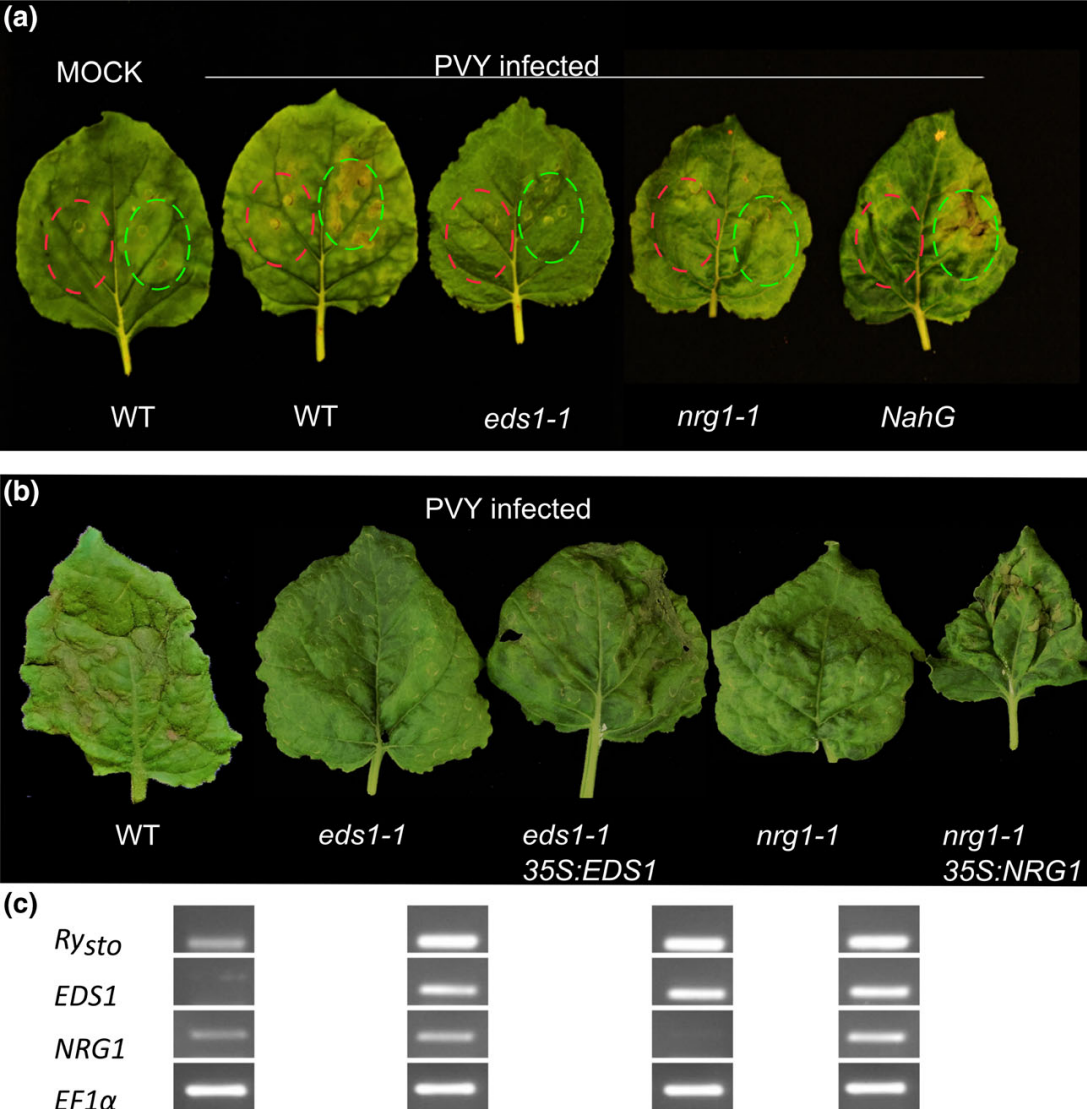
Downstream signalling components are crucial for *Ry*_*sto*_*‐*mediated immunity. (a) Fully developed leaves of *Nicotiana benthamiana eds1‐1* or *nrg1‐1* knockout plants, *NahG* plants or non‐transformed plants were infected with PVY^NTN^ or were mock treated with water. Two weeks later, leaves with symptoms of PVY infection were infiltrated with *A. tumefaciens* suspensions carrying pICSLUS 0001:*Ry*_*sto*_ (highlighted in green) or pGBT : *GFP* (highlighted in red) as a control. Three days after infiltration, symptoms of cell death were observed in non‐transformed plants and *NahG* plants ( SA ‐free). Experiments were repeated three times with similar results. (b) Complementation tests for *Ry*_*sto*_‐triggered HR in *eds1‐1* or *nrg1‐1 N. benthamiana* mutants. PVY ‐infected leaves of *N. benthamiana eds1‐1, nrg1‐1* knockouts or non‐transformed plants were infiltrated with *Agrobacterium* carrying *Ry*_*sto*_ alone or *Ry*_*sto*_ coexpressed with *EDS* *1* or *NRG* *1*. Three days after infiltration, HR was observed only when Ry_sto_ was coexpressed with indicated proteins. Experiments were repeated three times with similar results. Transcripts expression was confirmed via semi‐quantitative RT‐PCR (c) Elongation factor‐1α ( *EF* *1*α) was used as an internal control. Raw data are presented in the Figure S9.

It has been recently shown that TIR‐NLR signalling pathway may involve, in addition to Enhanced Disease Susceptibility 1 (EDS1), a well studied component, other helper proteins, including N requirement gene 1 (NRG1) (Castel *et al*., 2019; Qi *et al*., 2018). To check whether EDS1 and/or NRG1 are involved the *Ry*_*sto*_‐signalling, we assayed development of HR in response to PVY in *EDS1* and *NRG1* knockout background. To this aim, *Ry*_*sto*_ was transiently expressed in *eds1‐1* or *nrg1‐1 CRISPR/Cas9‐*mediated *N. benthamiana* mutant plants (Castel *et al*., 2019; Schultink *et al*.,^2017^) that had been pre‐inoculated with PVY^NTN^. While in the wild‐type control plants HR was observed 72 hpi, the *eds1‐1* and *nrg1‐1* plants remained symptomless (Figure 5a), suggesting that both EDS1 and NRG1 are required for Ry_sto_‐mediated response. Having shown that Ry_sto_‐mediated HR was abolished in *eds1‐1* and *nrg1‐1* lines, we then performed coexpression of Ry_sto_ either with EDS1 or NRG1 in the respective mutants to check whether HR phenotype will be restored. Consistently, expression of EDS1 and NRG1 restored the resistant phenotype in the mutants’ background (Figure 5b, c) implying that Ry_sto_ activates HR in EDS1‐/NRG1‐dependent manner. A similar analysis was performed with *N. benthamiana* plants expressing bacterial *NahG*, which encodes SA hydroxylase (Friedrich *et al*., 1995). Symptoms of HR were observed 72 hpi both in wild‐type and *NahG* plants (Figure 5a), indicating that elevated levels of SA were not essential for establishing *Ry*_*sto*_‐dependent resistance.

## Discussion

Genes conferring ER against PVY infection were previously introduced into potato cultivars from wild or domesticated species of the *Solanaceae* family, including *S. chacoense*,* S. stoloniferum* and *S. tuberosum* ssp*. andigena*. Two alleles of the *Ry*_*sto*_ gene derived from *S. stoloniferum* (*Ry*‐*f*_*sto*_ and *Ry*_*sto*_) have been used in breeding programmes (Flis *et al*., 2005; Song *et al*., 2005). In this study, we report the isolation and characterization of a novel, broad‐spectrum resistance *Ry*_*sto*_/*Ry‐f*_*sto*_ gene using RenSeq (Jupe *et al*., 2013; Witek *et al*., 2016).

We hypothesized that the underlying PVY resistance gene encoded a NLR protein. Data obtained from SMRT and Illumina sequencing together with SNP calling and presence/absence polymorphism detection revealed 11 transcriptionally active NLR genes that were candidates for the *Ry*_*sto*_ gene. These candidates mapped to the DM reference genome (Potato Genome Sequencing Consortium (95 authors), 2011) at the distal end of chromosome XII, in a region known to carry *Ry*_*sto*_‐linked markers. Previous research using graphical genotyping with a panel of tetraploid potatoes suggested that *Ry*_*sto*_ was located on superscaffold DMB114 (Van Eck *et al*., 2017). This was consistent with our data, as 3 of the 11 candidates were located on this superscaffold and the remaining candidates were located within 1 Mb, on neighbouring superscaffolds. All the linked NLRs showed presence/absence polymorphisms with the same allele ratios, indicating that the whole interval introgressed from *S. stoloniferum* and rarely undergoes recombination in cultivated potato.

Functional assays in *N. benthamiana* and cultivated potato plants confirmed that only one candidate, *c630*, prevented PVY multiplication and spread, hereafter termed *Ry*_*sto*_. The protein encoded by *Ry*_*sto*_ had motifs and domains typical of TIR‐NLR‐type resistance proteins and shared <34% identity at the amino acid level with previously described TIR‐NLR‐type resistance proteins from *Solanaceae*, including *N*,* Y‐1*,* Bs4* and *Pvr4*.

Sequence homologues of *Ry*_*sto*_ were amplified from eight PVY‐resistant cultivars (Barbara, Pirola, Ute, Wega, Assia, Fanal, Heidrun and Esta [for details, see Świeżyński *et al*., 1997; http://www.europotato.org]) from the collection at the Max Planck Institute for Plant Breeding Research (MPI‐PZ). No SNPs were observed in either the coding or the non‐coding sequences of the 4.85 kb amplified regions.

Most potato cultivars lack broad‐spectrum resistance to circulating strains of PVY (Valkonen, 2015). Recently, two novel genes (*Pvr4* and *TPN1*) were reported that conferred immunity to some PVY strains. Kim *et al*. (2017) isolated *Pvr4*, a dominant gene conferring a broad range of resistance to potyviruses, including some PVY isolates, from *Capsicum annuum*. However, *Pvr4* was tested only in *N. benthamiana*, a heterologous system, and not in any *Solanaceae* crops. Therefore, there is no direct evidence that *Pvr4* would be functional in a potato background. Similarly, *TPN1*, a single recessive gene, was isolated and tested only in tobacco where it causes venial HR‐like necroses (Michel *et al*., 2018). Our results suggest that *Ry*_*sto*_‐mediated immunity is effective in potato and tobacco plants not only against different strains of PVY but also against the related virus PVA (Table S1, Figure 2a and S6). This is in contrast to *Rx*,* Rsv3* and *Sw*‐*5*, which mediate ER to other viruses and to which immunity‐breaking isolates have emerged (Brommonschenkel *et al*., 2000; Moreira *et al*., 1980; Querci *et al*., 1995; Zhang *et al*., 2012).

Multiple PVY ORFs were tested, and viral CP was found to be the recognized in Ry_sto_‐mediated immunity against PVY. Similarly, CP was the elicitor of Ry_sto_‐mediated immunity for PVA (Figure 3a). The PVY and PVA CPs share ~59% identity (Figure 3b), and both display nucleocytoplasmic localization in infected cells (Figure 3d). The mechanism of CP recognition by Ry_sto_ remains unknown. As was previously shown for Rx, recognition may rely on a three‐dimensional structure containing a specific internal amino acid motif (Baurès *et al*., 2008; Candresse *et al*., 2010).

Rx induced HR when transiently expressed with PVX CP in a heterologous *N. benthamiana* system (Bendahmane *et al*., 1999). Similarly, PVY infection of *N*. *benthamiana* leaves transiently overexpressing *Ry*_*sto*_ produced a strong HR reaction (Figure 1c). HR was also elicited when PVY CP was transiently produced under control of a constitutive *35S* promoter in transgenic *Ry*_*sto*_ potato plants. By contrast, CP produced during PVY infection led to ER, indicating that the level of CP determined the type of response. However, when *Ry*_*sto*_ was introduced into *N. tabacum*, some necrotic lesions on inoculated leaves of resistant plants were observed upon PVY infection (Figure S3). This led us to the conclusion that the nature of the resistance response (ER or HR) is determined not only by the CP level but also by host factors differing between the plant models used. Modulation of the specificity of elicitor recognition was also observed for the *Rx* gene between PVX CP in *Rx*‐expressing potato and tobacco plants (Baurès *et al*., 2008).

ER or HR formation and control of viral spread might be determined by R gene expression level for different resistance genes. High levels of *HRT* transcript in susceptible *Arabidopsis thaliana* led to restriction of virus replication or movement without any visible symptoms of HR, whereas lower *HRT* transcript levels in resistant *Arabidopsis* lines led to micro‐HR or HR with systemic virus movement (Cooley *et al*., 2000). By contrast, our results suggest that ER is not affected by *Ry*_*sto*_ expression levels. Transgenic potato lines with *Ry*_*sto*_ under the control of native regulatory elements exhibited much lower transcript levels than lines with *Ry*_*sto*_ under the control of *35S*. ER phenotypes were observed throughout all the experiments regardless of relative *Ry*_*sto*_ expression levels (Figure 2b and 2d).

Here, we showed that the *Ry*_*sto*_ mode of action was, unusually for a TIR‐NLR‐type receptor, temperature‐independent (Figure 4b and 4c). Products of many *R* genes are non‐functional at or above 28 °C. Elevated temperatures reduced functionality for the *N* gene conferring resistance to TMV, the *Mi‐1* gene responsible for resistance to root‐knot nematodes (Hwang *et al*., 2000; Jablonska *et al*., 2007), the *Ny‐1* gene conferring resistance to PVY (Szajko *et al*., 2014), the suppressor of NPR1, constitutive 1 (*SNC1*) gene in *Arabidopsis* contributing to the resistance to the bacterial effector AvrRps4 (Yang and Hua, 2004) and many others. All these genes induced defence at permissive temperatures but experienced arrested HR development upon shifts to an elevated temperature. By contrast, *Ry*_*sto*_‐mediated immunity remained effective in different *Solanaceae* species at or above 28 °C (Figure 4b and 4c). Temperature‐sensing characteristics have also been identified in Arabidopsis *SNC1*, a NB‐LRR‐type gene closely related to *RPP4* and *RPP5*. Genetic screens of *snc1* mutants identified a single mutation (Gly380 to Ser) in the encoded protein that reverted the heat‐stable resistance response (Zhu *et al*., 2010). This provides evidence that temperature sensitivity of R genes might be controlled internally rather than by other regulatory components. In the Ry_sto_ protein, glycine is present at position 380, immediately after the putative GLPL motif in the NB‐ARC domain. This motif was previously identified as important for nucleotide binding, and mutations in residues close to this motif might compromise activation of NB‐LRR proteins (Rairdan and Moffett, 2006). It remains to be determined whether elements of the Ry_sto_ structure are responsible for its temperature stability.

A detailed model of the functional relationship between HR and ER has not yet been proposed. In principle, the products of gene expression for ER act earlier and more efficiently than the genes for HR, as demonstrated in comparative studies of potato *Rx* and *N* genes (Bendahmane *et al*., 1999). *Ry*_*sto*_ *‐*mediated ER was epistatic to *Ny‐1*‐mediated HR against PVY in potato (Figure 4).

The epistatic *Ry*_*sto*_ */Ny‐1* interaction was also observed in progeny of a cross between cv Rywal and clone PW 363 (Szajko *et al*., 2018). In contrast to *Ny‐1* plants (Baebler *et al*., 2014; Szajko *et al*., 2008), *Ry*_*sto*_ */Ny‐1* plants were PVY‐free at elevated temperature (28 °C). Collectively, *Ry*_*sto*_‐mediated ER was epistatic to HR. Nevertheless, the ER and HR pathways might be interdependent. For instance, Ry_sto_ might compete with Ny‐1 for recruitment of some signalling components. Assuming that Ry_sto_ has higher affinity to selected components than Ny‐1, *Ry*_*sto*_‐mediated immunity is stronger and progresses faster. These components might act as helper NLRs since many R proteins assemble into multi‐subunit complexes (Wu *et al*., 2018).

To activate immunity, most TIR‐NLRs require EDS1 protein (Aarts *et al*., 1998; Wiermer *et al*., 2005). Recently, it has been shown that also safeguarding proteins like activated disease resistance 1 (ADR1) and NRG1 may be involved in NLRs activation upon pathogen perception (Castel *et al*., 2019; Wu *et al*., 2018). In this study, we found that Ry_sto_ failed to initiate HR in *eds1* and *nrg1* knockout plants (Figure 5a, b). The HR phenotype was rescued by expression of EDS1 or NRG1 in the respective mutant background, consistent with the model, that Ry_sto_‐mediated resistance depends on EDS1 and NRG1. Thus, our results provide corroborating evidence for a critical role of NRG1 in the TIR‐NLRs immune signalling pathway (Castel *et al*., 2019; Qi *et al*., 2018). In our studies, HR in response to PVY infection was established in SA‐depleted *NahG* plants transiently expressing *Ry*_*sto*_ (Figure 5a). This indicates that, in contrast to EDS1, an increased SA level is not essential for *Ry*_*sto*_‐HR development. These data support a model that EDS1 performs different functions during plant immunity and the role played in triggering local cell death is independent of SA (Bhandari *et al*., 2019; Rietz, 2011).

Taking our observations as a whole, we propose a model in which *Ry*_*sto*_‐mediated immunity to ER against PVY results in *Ry*_*sto*_ recognition of PVY CP and recruitment of downstream signalling components such as *NRG1* and *EDS1*. Since *ADR1* activity may also contribute to TIR‐NLR‐mediated immunity (Dong *et al*., 2016), we cannot exclude *ADR1* involvement in the *Ry*_*sto*_ mode of action. Conceivably, co‐chaperone complex components SGT1, RAR1 and HSP90 might also be involved in *Ry*_*sto*_‐mediated immunity through their roles in supporting correct folding of resistance proteins (Peart *et al*., 2005). *Ry*_*sto*_ might also be negatively regulated through miRNA activity through promotion of mRNA degradation, inhibition of translation or suppression of transcription by epigenetic modification (Križnik *et al*., 2017).

In summary, our results demonstrate that *Ry*_*sto*_ plays an important role in defence and may prove valuable for breeding PVY‐resistant cultivars of potato and other *Solanaceae* crops. It remains unclear why some NLRs trigger ER and others trigger HR, but ER recognition appears to produce durable and efficient resistance.

## Experimental procedures

### Plant material

All potato cultivars (*S. tuberosum* ssp. tuberosum) and viruses PVY^0^, PVY^N^, PVY^N‐Wi^, PVX and PVA were obtained from the Laboratory for Potato Gene Resources and In Vitro Cultures at the Institute of Plant Breeding and Acclimatization – National Research Institute, Bonin, Poland. The cultivar Alicja (the source of *Ry*_*sto*_) was released from the breeding company HZ Zamarte, Poland, as a cross between clone OL‐21852 and cv. Ora (Figure S8). A *S. stoloniferum* accession from the collection of the Max Planck Institute for Plant Breeding Research (MPI‐PZ), Cologne, Germany, was in the ancestry of OL‐21852. PW363 (the source of *Ry‐f*_*sto*_) was generated in the parental line breeding programme at the IHAR‐PIB, Młochów. The ancestry of PW363 contained a *S. stoloniferum* accession from the collection of the Vavilow Research Institute of Plant Industry (VIR), St. Petersburg, Russia. Cultivars, Barbara, Pirola, Ute, Wega, Assia, Fanal, Heidrun and Esta from MPI‐PZ collection, were obtained from the Laboratory for Potato Gene Resources and in Vitro Cultures at the IHAR‐PIB. Plants were grown for 4 weeks in soil under controlled environmental conditions (22 °C, 16‐h light; 18 °C, 8‐h dark) as described previously (Szajko *et al*., 2008). Tobacco plants *Nicotiana tabacum* cv. Xanthi‐nc and *N. benthamiana* were grown for 6 weeks in soil under controlled environmental conditions (22 °C; 16‐h light and 8‐h dark) as described previously (Hoser *et al*., 2013). Transgenic potato and tobacco plants were regenerated following Agrobacterium leaf disc transformation as described by Mac *et al*. (2004).

### Mapping population

A diploid potato (2*n *= 2*x *= 24) mapping population was developed from a cross between PVY‐resistant heterozygous clone dH Alicja and susceptible clone DW 83‐3121. dH Alicja was obtained from potato cultivar Alicja (Świerzyński *et al*., 1997; http://www.europotato.org) via parthenogenesis. ER to PVY in dH Alicja was conferred by the *Ry*_*sto*_ gene, which derived from clone MPI 55.957/54. The pedigree of MPI 55.957/54 included *S. stoloniferum* (Ross, 1958; *Handbuch der Pflanzenzüchtung*, 2nd, Vol. III:106‐125).

### Short‐ and long‐read RenSeq and bioinformatic analysis of sequencing data

Construction and enrichment of short‐read RenSeq libraries was carried out on susceptible (S, clone DW 83‐3121), resistant (R, dHAlicja) and 149 bulked susceptible (BS) plants as described previously (Jupe *et al*., 2013; Witek *et al*., 2016). Enriched libraries were sequenced with an Illumina MiSeq platform using 500 cycles. RenSeq was also performed on cDNA from resistant parents as described previously (Witek *et al*., 2016) and sequenced using an Illumina HiSeq platform with 300 cycles. Long‐read PacBio RenSeq was performed as described in Witek *et al*. (2016) and sequenced using a single PacBio RSII SMRT cell, resulting in 69,737 ROIs with a minimum of three passes and accuracy >90% (available at accession number MN393235). Reads obtained from SMRT RenSeq were assembled using Geneious 8.1.2 software as described previously (Witek *et al*., 2016), resulting in 1555 contigs. R, S and BS reads from Illumina MiSeq 250PE sequencing were mapped to contigs derived from the SMRT RenSeq assembly using BWA (Li and Durbin, 2009), with default settings. SNP calling and candidate prediction were performed as described previously (Jupe *et al*., 2013; Witek *et al*., 2016). Contigs with >95% of susceptible alleles in mapped BS data were considered to be linked. Candidate NLRs showing presence/absence polymorphism between the R and S parents and linkage to resistance based on BS samples were also called. Briefly, numbers of pair‐end mapped reads relative to contigs derived from the SMRT RenSeq assembly were calculated for R, S and BS samples using TSL Galaxy built‐in scripts (Maclean and Kamoun, 2012). The resulting data were sorted and visualized using Microsoft Excel software. To define presence/absence polymorphism, contigs with at least 250 mapped reads from the R parent, and <10% and 18% of that number for S and BS samples, respectively, were considered. Expression of the candidate genes was determined using cDNA‐RenSeq data from the R parent as described previously (Andolfo *et al*., 2014; Witek *et al*., 2016).

### DNA extraction

RenSeq and PCR were performed on freshly extracted genomic DNA obtained from young leaves using a DNeasy Plant Mini Kit (Qiagen, Hilden, Germany) according to the manufacturer's protocol.

### RNA extraction and cDNA preparation for RenSeq

For cDNA‐RenSeq, RNA was extracted using TRI‐Reagent (Sigma‐Aldrich, Saint Louis, MO) and a Direct‐zol RNA MiniPrep Kit (Zymo Research, Tustin, CA) according to the manufacturers’ instructions. First‐strand cDNA was synthesized using a mix of oligo‐dT and random hexamer primers and the SuperScript II First‐Strand Synthesis System (Sigma‐Aldrich). Second‐strand cDNA was synthesized as described previously (Rallapalli, 2014).

### Cloning of candidate ORFs

PCR primers flanking predicted ORFs were designed for each of 12 selected PacBio contigs. Specific 5′ and 3′ extensions were added to primers for compatibility with the custom USER expression vectors used in this study and described previously (Witek *et al*., 2016). Candidate genes were PCR‐amplified from R parent genomic DNA in 25 μL PCR reactions (35 cycles with annealing at 62 °C and 7 min extension at 72 °C) using Kappa HiFI HotStart Uracil+ Fidelity Polymerase (Manufacturing, R&D Cape Town, South Africa). Purified PCR product (30 ng) was hybridized with 30 ng pICSLUS0003 vector in the presence of 1 μL USER enzyme mix (New England Biolabs, Inc., Ipswich, MA) as described previously (Witek *et al*., 2016). All constructs were verified by DNA sequencing. Plasmids containing candidate genes were transformed into *Agrobacterium* strain GV3101 for transient complementation assays or strain LBA404 for stable potato transformation using the method described previously (Mac *et al*., 2004). To create a construct containing *Ry*_*sto*_ (*c630*) under the control of its native regulatory elements, the whole contig (7.5 kB) assembled from PacBio reads was PCR‐amplified and introduced into USER‐vector pICSLUS0001 lacking the *35S* promoter and OCS terminator. List of primers used in this study is enclosed as Table S5.

### Transient expression assay

*Agrobacterium* GV3101 strains carrying plasmids containing the candidate genes or PVY‐derived sequences were used to infiltrate *N. benthamiana*. Bacteria harbouring plasmids were grown in YEB medium supplemented with gentamicin, rifampicin and kanamycin. Overnight cultures were collected by centrifugation at 3500 rpm, resuspended in infiltration buffer containing 10 mm MgCl_2_, 10 mM 2‐[N‐morpholino] ethanesulfonic acid (MES) (pH 6.5) and 100 μm acetosyringone to a final OD_600_ = 1 and infiltrated into leaves of 4‐week‐old plants using a 3 mL syringe. HR reactions were observed 2–3 days after infiltration. Each experiment was performed at least twice and included at least three independent biological replicates.

### Resistance assay

PVY isolate NIB‐NTN (GenBank: AJ585342.1) was used in all experiments involving PVY infection monitoring. Seven‐week‐old *N. tabacum* plants or 4‐week‐old potato plants were infected under the previously described conditions (Baebler *et al*., 2014). Where different virus isolates were used, plants were infected under the same experimental conditions with PVY isolates 0 (AJ890349), N (FJ666337) or N‐Wilga (EF558545), or unrelated viruses PVA, PVX or TMV(U1).

### Semi‐quantitative RT‐PCR

Total RNA was treated with DNase I (Thermo Fisher Scientific, Waltham, MA) and subjected to reverse transcription using a mix of random hexamers and oligo‐dT primers and a RevertAid First‐Strand cDNA Synthesis Kit (Thermo Fisher Scientific). Semi‐quantitative RT‐PCR was performed using DreamTaq (Thermo Scientific) with 25 to 30 amplification cycles followed by electrophoresis with 2% agarose gel stained with Ethidium bromide. Primer pairs used in the PCR reaction are listed in Table S6.

### Gene expression analysis‐ RT‐qPCR

Gene expression analysis via RT‐qPCR was performed using a LightCycler^®^480 instrument and LightCycler^®^480 SYBR Green I Master Kit reagents (Roche, Indiana, IN). Relative gene expression levels were determined using a standard curve method, and the value for each target gene was normalized against the mean of expression values of two reference genes, *EF1* and *L23* for *N. tabacum* and *N. benthamiana* or *EF1* and *Sec3* for potato cultivars, as described previously (Liu *et al*., 2012; Tang *et al*., 2017). Each sample was tested with four technical replicates and two dilutions. Primers for qPCR are provided in Table S6.

### PVY ORFs cloning

A full‐length infectious PVY‐N605 clone (Jakab *et al*., 1997) was used as a template to create all PVY constructs. ORFs encoding each putative PVY protein were PCR‐amplified and cloned into the pBIN53‐mCherry vector with a CaMV *35S* promoter, a polylinker separating the PVY 5′ and 3′ UTRs and a poly(A) tail as described previously (Mestre *et al*., 2000).

### PVA CP cDNA isolation

PVA CP encoding cDNA was amplified by reverse transcription‐PCR from total RNA extracted from PVA‐infected tobacco leaf tissue. The PCR fragment was inserted into the pENTR/D‐TOPO vector (Invitrogen, Carlsbad, CA). The resulting entry clones were LR recombined with the Gateway pGWB 454 destination vector (Nakagawa *et al*., 2007).

### Western blotting

*Agrobacterium*‐infiltrated leaves were collected, frozen and ground in liquid nitrogen. Total proteins were extracted by incubating ground leaf samples in extraction buffer containing 100 mm Tris HCl pH 8.0, 1 mm EDTA, 150 mm NaCl, 7.2 mm β‐mercaptoethanol, 0.5 mm 4‐(2‐aminoethyl) benzenesulfonyl fluoride hydrochloride (AEBSF) and 0.03 μm PMSF (phenylmethylsulfonyl fluoride) for 15 min. Homogenates were centrifuged at 21,000 ***g*** for 10 min, and the supernatants were collected. Samples were separated by 12.5% SDS‐PAGE and subjected to immunoblot analysis using polyclonal Anti‐mCherry antibody (Abcam, Cambridge, UK) or appropriate alkaline phosphatase‐conjugated anti‐PVA antibodies (Bioreba, Reinach, Switzerland). Immunoblots were developed using a NBT/BCIP colorimetric detection kit (BioShop Inc., Canada).

### Confocal laser scanning microscopy

Subcellular localization of the fusion proteins was evaluated using a Nikon C1 confocal system built on TE2000E and equipped with a 60×Plan‐Apochromat oil immersion objective (Nikon Instruments B.V. Europe, Amsterdam, The Netherlands). RFP fusion protein was excited by a 543 nm helium‐neon laser and detected using the 605/75 nm barrier filter. Confocal images were analysed using free viewer EZ‐C1 and ImageJ software.

### Statistical analysis

Statistical analyses were conducted using R 3.2.2 within R Studio 0.99.483. Technical replicates consisted of replicate readings from the same plant in the same experiment, whereas biological replicates consisted of measurements obtained from independent plants. Data were analysed using the following pipeline: data were assessed for their suitability for parametric analysis by testing for the normal distribution of the residuals using a Shapiro–Wilk test. If the data were suitable for conducting parametric tests, then analysis of variance (ANOVA) was used. Dunnett's or Tukey's HSD (*P *< 0.001**) tests were used for post hoc analysis.

## Author contributions

M.G‐B., K.W and J.H. performed most of the experimental work, data analyses and writing; A. W., I.W‐F., H.J., K.S. and K.M performed the research; J.J. and W.M. edited the article; J.H. supervised the work.

## Competing interests

KW, MGB, JH, JDGJ, WM and KS have filed a US patent application 62/538.020 based on this work.

## Supporting information


**Figure S1** Phylogenetic analysis of *Ry*_*sto*_ candidate genes and other functional *Solanaceae* NLRs.
**Figure S2** Grafted transgenic *Ry*_*sto*_ plants display resistance to PVY.
**Figure S3** HR of transgenic *N. tabacum* expressing *Ry*_*sto*_.
**Figure S4** Stable transgenic tobacco plants carrying *Ry*_*sto*_ under the control of a 35S promoter display resistance to PVY. Seven‐week‐old *N. tabacum 35S:Ry*_*sto*_ transgenic and non‐transformed plants were inoculated with PVY^NTN^ or mock treated with water.
**Figure S5** Stable transgenic tobacco plants carrying *Ry*_*sto*_ under the control of native regulatory elements display resistance to PVY. Seven‐week‐old *N. tabacum Ry*_*sto*_ (native) transgenic and non‐transformed plants were inoculated with PVY^NTN^.
**Figure S6** *Ry*_*sto*_‐mediated resistance to PVA infection.
**Figure S7** Comparison of *Ry*_*sto*_ and *Ry‐f*_*sto*_ nucleotide sequences.
**Figure S8** Pedigree chart of cultivar Alicja.
**Figure S9** Semi‐quantitative RT‐PCR of *Ry*_*sto*_, *NRG1* and *EDS1* genes.

**Table S1** PVY dependent HR in *N. benthamiana* plants expressing *contig 630*.
**Table S2** ELISA titers of PVY in transgenic *35S::Rysto S. tuberosum* cv. Maris Piper plants.
**Table S3** ELISA titers of PVY in transgenic *35S::Rysto S. tuberosum* cv. Russet Burbank plants.
**Table S4** ELISA titers of PVY in grafted transgenic *35S::Rysto S. tuberosum* cv. Maris Piper plants.
**Table S5** Primers used to clone candidate *Rysto* genes.
**Table S6** Primer sequences for quantitative RT and qRT‐PCR
