# Supplementary material for: Extreme resistance to Potato virus Y in potato carrying the Ry sto gene is mediated by a TIR‐NLR immune receptor
Source: Plant Biotechnol J. 2019 Sep 4;18(3):655–67. doi: 10.1111/pbi.13230 (PMC7004898; doi:10.1111/pbi.13230)
Supplement: Supplementary file 1 — Figure S1 Phylogenetic analysis of Ry sto candidate genes and other functional Solanaceae NLRs. Figure S2 Grafted transgenic Ry sto plants display resistance to PVY. Figure S3 HR of transgenic N. tabacum expressing Ry sto . Figure S4 Stable transgenic tobacco plants carrying Ry sto under the control of a 35S promoter display resistance to PVY. Seven‐week‐old N. tabacum 35S:Ry sto transgenic and non‐transformed plants were inoculated with PVYNTN or mock treated with water. Figure S5 Stable transgenic tobacco plants carrying Ry sto under the control of native regulatory elements display resistance to PVY. Seven‐week‐old N. tabacum Ry sto (native) transgenic and non‐transformed plants were inoculated with PVYNTN. Figure S6 Ry sto ‐mediated resistance to PVA infection. Figure S7 Comparison of Ry sto and Ry‐f sto nucleotide sequences. Figure S8 Pedigree chart of cultivar Alicja. Figure S9 Semi‐quantitative RT‐PCR of Ry sto , NRG1 and EDS1 genes. [file PBI-18-655-s002.pdf]

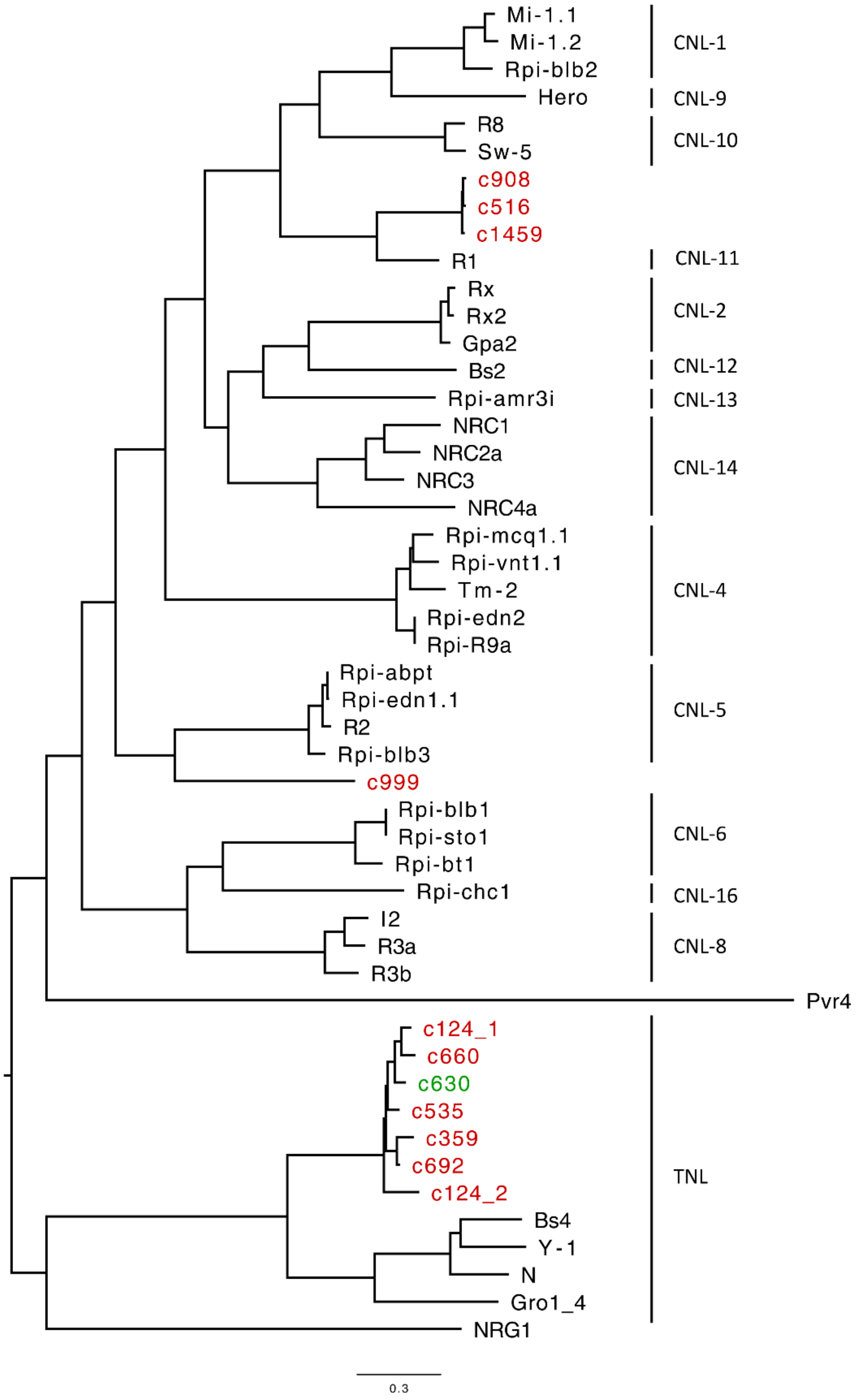

Supporting Figure 1 Phylogenetic analysis of *Ry*$_{sto}$ candidate genes and other functional *Solanaceae* NLRs. Full-length amino acid sequences were aligned using ClustalW 1.74. Evolutionary analyses were conducted using MEGA6.06 using the Maximum-Likelihood statistical method with 100 bootstraps and Poisson (G+I) model for amino acid substitutions.

Tree represents typical Solanaceae NLR architecture. Clades are named according to Andolfo *et al*. (2014) and Witek *et al*. (2016). Candidate contigs are highlighted in red (c630) and green (all remaining). Sequences of functional NLRs were retrieved from public databases; Mi-1.1: AF039681, Mi-1.2: AF039682, Rpi-blb2: DQ122125, Hero: AX337980, R8: ANJ02805, Sw-5: AY007366, R1: AF447489, Rx: AJ011801, Rx2: CAB56299, Gpa2: AAF04603, Bs2: AF202179, Rpi-amr3i: KT373889, NRC1: DQ304484, NRC2a: KT936525, NRC3: XM_004238900, NRC4a: Solyc04g007030, Rpi-mcq1.1: WO2009013468, Rpi-vnt1.1: FJ423044, Tm-2: AY742887, Rpi-edn2: MI377464, Rpi-R9a: AVX67693, Rpi-abpt: FJ536324, Rpi-edn1.1: GU563963, R2: FJ536325, Rpi-blb3: FJ536326, Rpi-blb1: FB764493, Rpi-sto1: EU884421, Rpi-bt1: FJ188415, Rpi-chc1: WO2011034433, I2: AF118127, R3a: AY849382, R3b: JF900492, Pvr4: KT359375, Bs4: AY438027, Y-1: CAC82812, N: Q40392, Gro1-4: AAP44390 and NRG1: AAY54606.

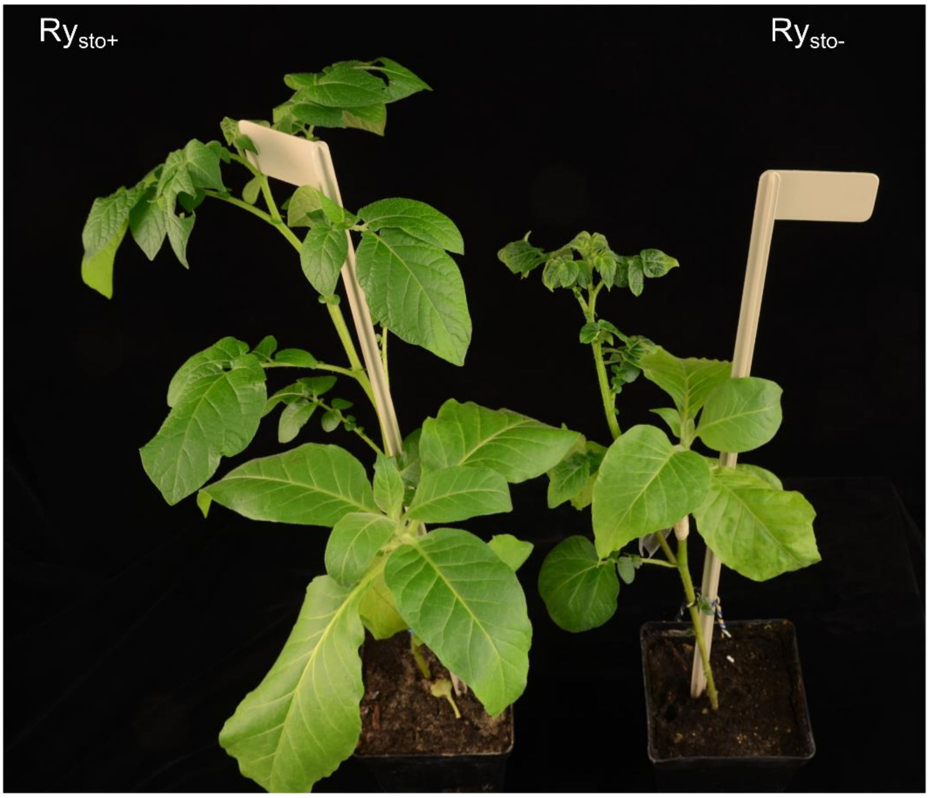

Supporting Figure 2 Grafted transgenic *Ry*$_{sto}$ plants display resistance to PVY. Scions (upper part) of a non-transformed *N. tabacum* plants that had been preinoculated with PVY^{NTN} were grafted to a stock (lower part) of stable transgenic *35S:Ry*$_{sto}$ Maris Piper or susceptible to PVY Nicola plants. Three weeks after grafting chlorosis and dwarfing were observed only in susceptible plants (right) whereas *Ry*$_{sto}$ transgenic plants (left) remained symptomless. The experiment was performed on three independent transgenic potato lines and three biological replicates were used for each line. Picture was taken 4 weeks after grafting.

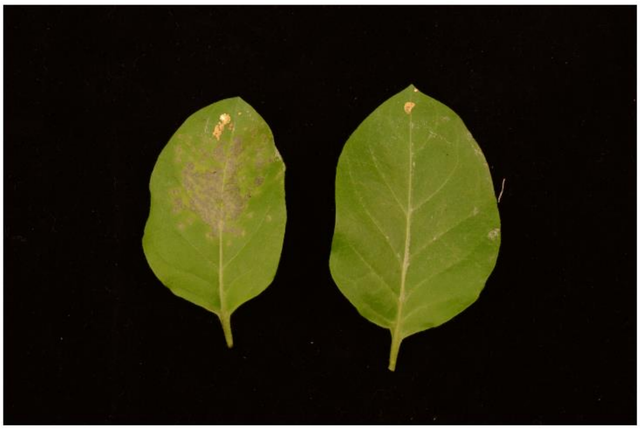

Supporting Figure 3 HR of transgenic *N. tabacum* expressing *Ry*$_{sto}$. HR response after PVY inoculation in stable transgenic *N. tabacum* plants transformed with *35S:Ry*$_{sto}$. Seven-week-old *N. tabacum 35S* transgenic and non-transformed plants were inoculated with PVY^{NTN}. HR was observed on *N. tabacum 35S:Ry*$_{sto}$ inoculated leaves at 3 dpi, whereas control plants remained symptomless. Image was taken at 7 dpi.

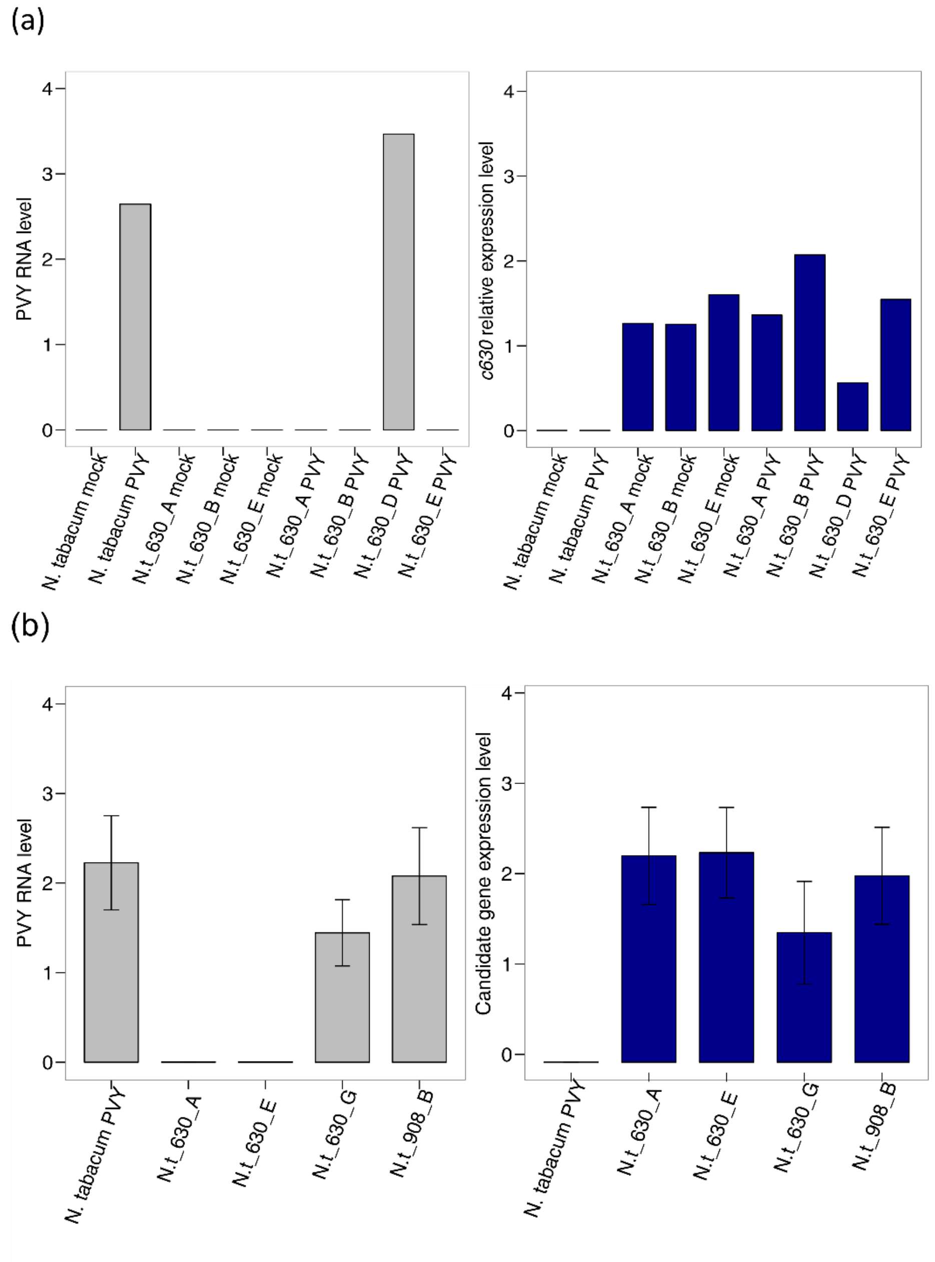

Supporting Figure 4 Stable transgenic tobacco plants carrying *Ry*$_{sto}$ under the control of a 35S promoter display resistance to PVY. Seven-week-old *N. tabacum 35S:Ry*$_{sto}$ transgenic and non-transformed plants were inoculated with PVY^{NTN} or mock treated with water. Two weeks after PVY inoculation, mRNA was isolated from upper, non-inoculated leaves and quantified with qPCR. (a) Elevated expression of *Ry*$_{sto}$ correlated with lack of PVY RNA (lines A, B and E) in stable T0 transgenic plants. (b) Resistant lines A and E (T1 generation) confirmed T0 observations. Line 630G (susceptible), where expression of *Ry*$_{sto}$ was not

detected, had PVY RNA levels similar to a control line (*c908B*) carrying a non-functional paralogue.

(a)

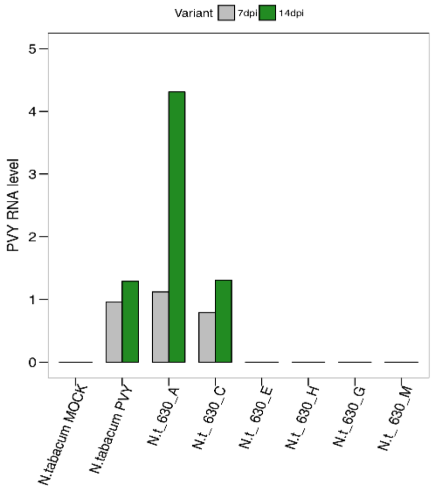

(b)

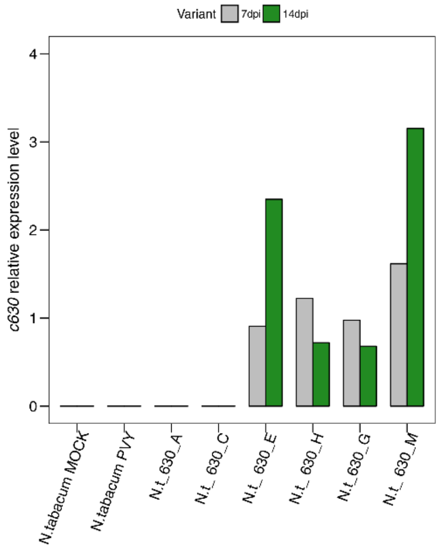

Supporting Figure 5 Stable transgenic tobacco plants carrying *Ry*$_{sto}$ under the control of native regulatory elements display resistance to PVY. Seven-week-old *N. tabacum Ry*$_{sto}$ (native) transgenic and non-transformed plants were inoculated with PVY^{NTN}. Seven and fourteen days after PVY inoculation, mRNA was isolated from upper, non-inoculated leaves and measured using qPCR. *EF1* and *L23* were used as standardisation reference genes. In lines E, G, H and M (T0 generation) the inhibition of virus multiplication (a) correlated with a high level of *Ry*$_{sto}$ transcript (b).

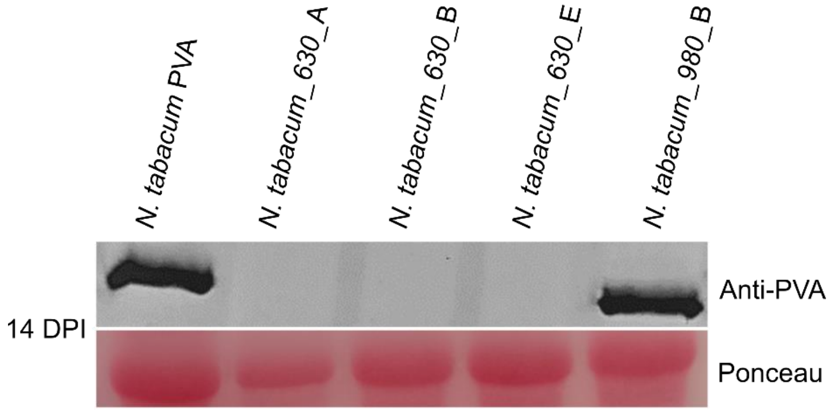

Supporting Figure 6 *Ry*$_{sto}$-mediated resistance to PVA infection. Western blot analysis of PVA accumulation in leaves of transgenic *Ry*$_{sto}$ and non-transformed *N. tabacum* plants after 14 days. Seven-week-old *N. tabacum 35S:Ry*$_{sto}$ or *35:c908B* (control line carrying a non-functional paralogue) and non-transformed plants were inoculated with PVA. Two weeks after infection, samples of upper, non-inoculated leaves were harvested and subjected to immunoblot analysis using alkaline phosphatase-conjugated anti-PVA antibodies.

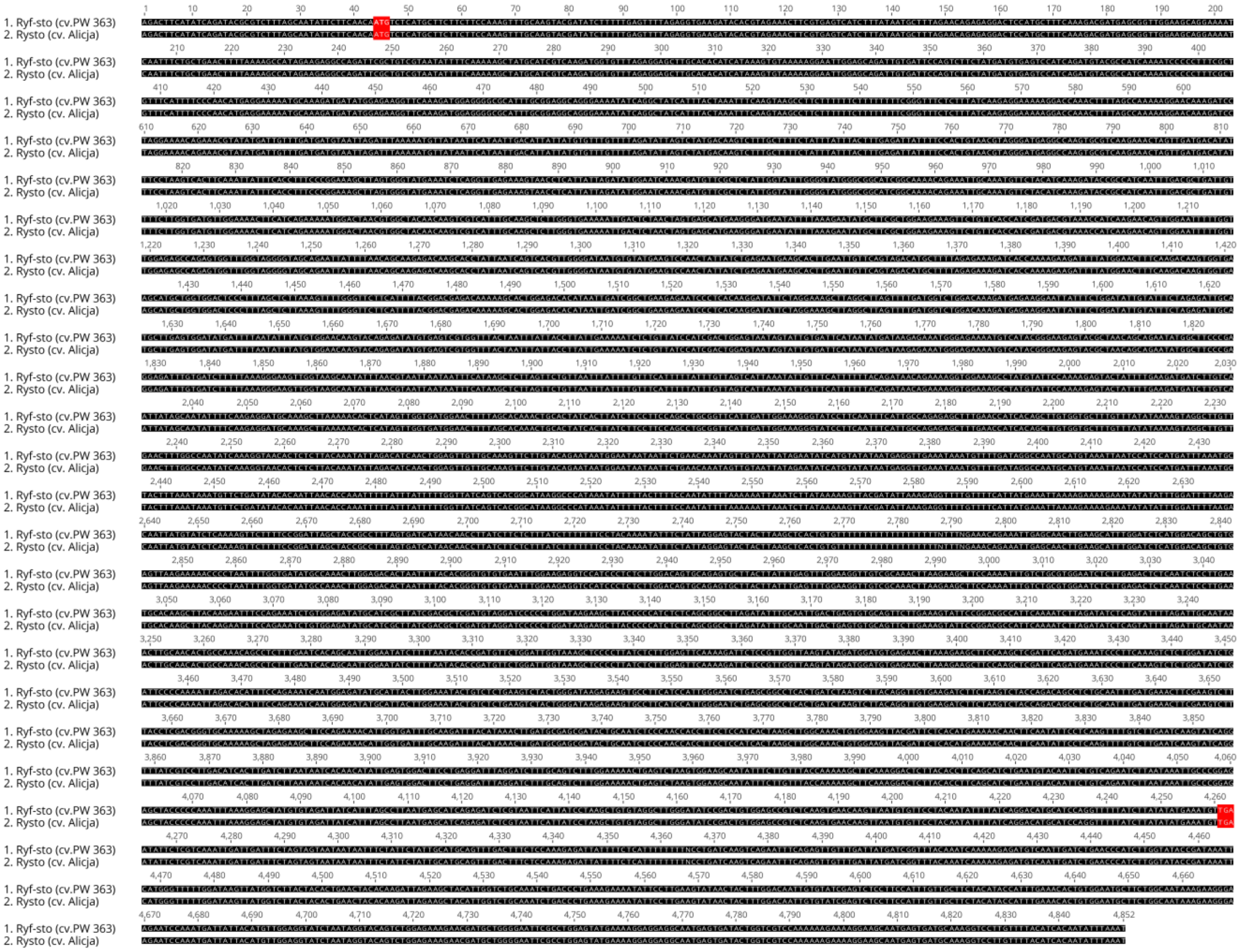

Supporting Figure 7 Comparison of Ry_{sto} and $Ry\text{-}f_{sto}$ nucleotide sequences. Identical residues are shaded in black. Start and Stop codons are marked with red. The alignment was generated using MAFFT-L-INS-I (Katoh & Toh, 2008) and visualised in Jalview 2,10,4b1 (Waterhouse et al., 2009).

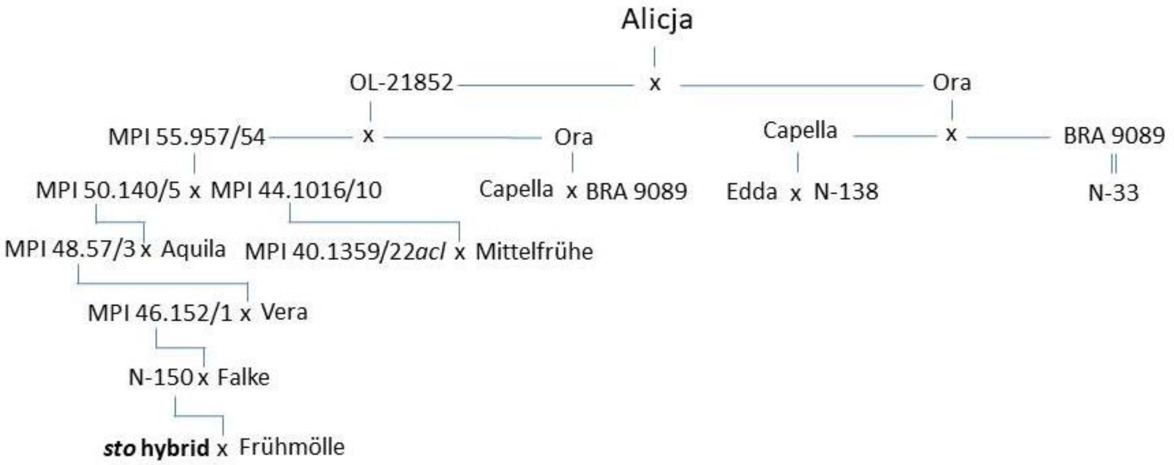

Supporting Figure 8 Pedigree chart of cultivar Alicja. Cultivar Alicja was obtained from a cross between clone OL-21852 and cultivar Ora at the breeding company HZ Zamarte, Poland. The pedigree chart was prepared according to the data described by Świerzyński *et al*. (1997). The Ry_{sto} gene source is in bold.

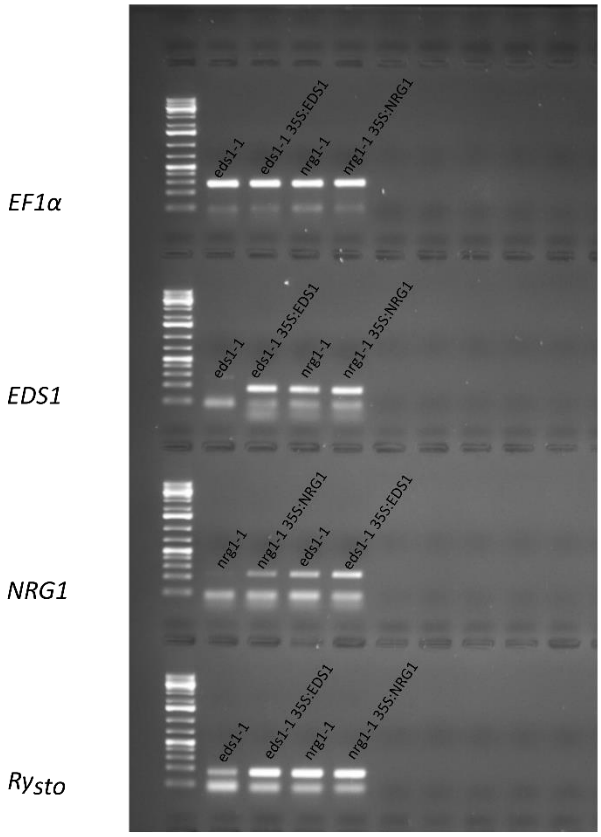

Supporting Figure 9 Semi–quantitative RT-PCR of *Ry*$_{sto}$, *NRG1* and *EDS1* genes. PVY-infected leaves of *eds1-1, nrg1-1 N. benthamiana* knockout plants were infiltrated with *Agrobacterium* carrying *Ry*$_{sto}$ alone or *Ry*$_{sto}$ coexpressed with *EDS1* or *NRG1*. Samples were collected two days after infiltration. Elongation factor-1α (*EF1*α) was used as an internal control.
