# Supplementary material for: Extreme resistance to Potato virus Y in potato carrying the Ry sto gene is mediated by a TIR‐NLR immune receptor
Source: Plant Biotechnol J. 2019 Sep 4;18(3):655–67. doi: 10.1111/pbi.13230 (PMC7004898; doi:10.1111/pbi.13230)
Supplement: Supplementary file 2 — Table S1 PVY dependent HR in N. benthamiana plants expressing contig 630. Table S2 ELISA titers of PVY in transgenic 35S::Rysto S. tuberosum cv. Maris Piper plants. Table S3 ELISA titers of PVY in transgenic 35S::Rysto S. tuberosum cv. Russet Burbank plants. Table S4 ELISA titers of PVY in grafted transgenic 35S::Rysto S. tuberosum cv. Maris Piper plants. Table S5 Primers used to clone candidate Rysto genes. Table S6 Primer sequences for quantitative RT and qRT‐PCR [file PBI-18-655-s001.pdf]

Supporting Table 1. PVY dependent HR in *N. benthamiana* plants expressing *contig 630*

Virus	*A. tumefaciens*/c630	*A. tumefaciens*/ -
0 (LW)	++	_
N (Ny)	++	_
N-Wilga	++	_
NTN (Slovenia)	++	_
PVX (0 strain)	_	_
TMV (U1)	_	_

The fully developed leaves of *N. benthamiana* plants were inoculated with a suspension of *A. tumefaciens* carrying pICSLUS0003-*c630*, or with *A. tumefaciens* without any plasmid as a control. Two weeks before the *Agrobacterium* infiltration the plants were infected with four different isolates of PVY(0, N, N-Wilga and NTN), PVA or other viruses as a control. Description: -no HR, +/- -weak HR, + HR, ++ strong HR (72 hpi). Experiment was repeated three times.

Supporting Table 2. ELISA titers of PVY in transgenic *35S::Ry$_{sto}$* *S. tuberosum* cv. Maris Piper plants.

Genotype	A_{405} values[a]
Maris Piper MOCK	0,035±003
Maris Piper PVY	1,957±0,22
MP_630_ A	0,016±0,017
MP_630_ B	0,010±0,004
MP_ 630_ C	0,005±0,001
MP_ 630_ D	0,004±0,002
MP_630_ E	0,009±0,003
MP_ 630_ F	1,713±0,093
MP_630_ G	0,013±0,003
MP_ 630_ H	1,896±0,188
MP_630_ I	0,030±0,029
MP_ 630_ J	0,011±0,001
MP_ 630_ K	0,022±0,007
MP_ 630_ L	0,015±0,009

[a]Mean ELISA absorbance values ± standard errors for three plants each. The plants were tested 3 weeks after inoculation with PVY^{NTN}. ELISA readings were taken after 2 h of incubation with substrate at room temperature as described by Syller (1991).

Supporting Table 3. ELISA titers of PVY in transgenic *35S::Ry_{sto}* *S. tuberosum* cv. Russet Burbank plants.

Genotype	A_{405} values[a]
Russet Burbank MOCK	0,046 ±0,002
Russet Burbank PVY	2,495±0,181
RB_630_A	0,006±0,000
RB_630_B	0,005±0,001
RB_630_C	0,005±0,001
RB_630_D	0,004±0,002

[a]Mean ELISA absorbance values ± standard errors for three plants each. The plants were tested 3 weeks after inoculation with PVY. ELISA readings were taken after 2 h of incubation with substrate at room temperature as described by Syller (1991).

Supporting Table 4. ELISA titers of PVY in grafted transgenic *35S::Ry$_{sto}$* *S. tuberosum* cv. Maris Piper plants.

Genotype	A_{405} values[a]
Nicola* MOCK	0,046 ±0,002
Nicola* PVY	3,303±0.278
MP_630_A	0,005±0,001
MP_630_N	0,005±0,0002
MP_630_R	0,005±0,001

[*] PVY-susceptible potato cultivar

[a]Mean ELISA absorbance values ± standard errors for three plants each. The plants were tested 3 weeks after inoculation with PVY. ELISA readings were taken after 2 h of incubation with substrate at room temperature as described by Syller (1991)

Supporting Table 5. Primers used to clone candidate Ry_{sto} genes

Gene	Primer	Sequence
c1241	124_1_35S_F	**GGCTTAAU**CTTTAGCAATATTCTTCAACAATG
	124_1_35S_R	**GGTTTAAU**TGACTGACATATGGTTCGTATATATC
c124_2	122_1_35S_F	**GGCTTAAU**GAGAAGTTACCTGTTAGTCTTTAG
	124_2_35S_R	**GGTTTAAU**AACATATTCTTTCCATACCATTTTATGATAC
c359	359_35S_F	**GGCTTAAU**CTGTTAGTCTTTAGCAATATTCTTCAACAATGG
	359_35S_R	**GGTTTAAU**CATGTTTGGTTGAATACAAAATTTAAGAAAGAC
c516	516_35S_F	**GGCTTAAU**CCTCTTTGCAGAAGCATTATAGATTCT
	516_35S_R	**GGTTTAAU**TGTCACGCTAAGGAGATTAAATTCTC
c535	535_35S_F	**GGCTTAAU**GGGTCTTCATTGCTGATAAAAATTCTG
	535_35S_R	**GGTTTAAU**GTGACTTAACACTTAATTTGAGTGTTAAATATTTTC
c630	630_35S_F	**GGCTTAAU**GGTGCTAAGAAGACTTCATATCAG
	630_35S_R	**GGTTTAAU**GGATACTCAACGTATCTACCTTATTATAA
c660	660_35S_F	**GGCTTAAU**GTATTGACTTGTCACACTTGTTAATTC
	660_35S_R	**GGTTTAAU**GGGATGAGGCTGAGTGCGTCAAG
c692	692_35S_F	**GGCTTAAU**CTGTTAGTCTTTAGCAATATTCTTCAACAATGG
	692_35S_R	**GGTTTAAU**CATGTTTGGTTGAATACAAAATTTAAGAAAGAA
c908	908_35S_F	**GGCTTAAU**CAAGGACTTTATGCATTGAACTTCATTCA
	908_35S_R	**GGTTTAAU**GTTCAACATGGATTCAATGTTACTCAATAC
c999	999_35S_F	**GGCTTAAU**ACGCCTAAATCTCCCTGAATAGTCTATT
	999_35S_R	**GGTTTAAU**GCAGATGAAGTAAACTAATGACAATTATAGTAT
c1459	1459_35S_F	**GGCTTAAU**GCAGAAGCATCACAGATTCTTTTAGTC
	1459_35S_R	**GGTTTAAU**GACAACAACTTTTACCGGTTACGTCAAAG
c630N	630_genomic_F1	**GGCTTAAU**CCAGGAAAACCTCGTCCATCAA
	630_genomic_R1	**AGCGTACTCU**TCCCGTATGAC ATT TTC TCC
	630_genomic_F2	**AGAGTACGCU**AACAGCAGAATATGGCTTCC
	630_genomic_R2	**GGTTTAAU**GGGCCAGAGAAAAAGTTGAGCAATCATC

In bold - extension for USER cloning

Normal font - gene specific sequence

35S - primers to clone gene into expression vector under 35S promoter and OCS terminator

genomic - primers to clone gene into expression vector under the control of native regulatory elements.

Supporting Table 6. Primer sequences for quantitative RT and qRT-PCR

Gene	Primer	Sequence
124_1	qPCR_124_1_35S_F	GCGAGCGATACTGCAATCTC
	qPCR_124_1_35S_R	ATCCTCAGGAAGTCCACTCAATATG
c124_2	qPCR_124_1_35S_F	GCGAGCAAAACTGCAATATACCA
	qPCR_124_2_35S_R	GATGCAAGACAAAACTTGAGGAATGT
c359	qPCR_359_35S_F	CGCACTTTTAAAGATGATGAGCG
	qPCR_359_35S_R	GGTGTTTAGAGGAGCTTGCACACATC
c516	qPCR_516_35S_F	GCTCACTTTGAAGAATGGTCTG
	qPCR_516_35S_R	TCTCCTTAAGAAATTTGCAATCCC
c535	qPCR_535_35S_F	TGTCTGCGTGGAATCTCTTG
	qPCR_535_35S_R	CTACATCGAGCGTCGATAAGC
c630	qPCR_630_35S_F	ATGCTTTCAAAGACGATGAGCGGT
	qPCR_630_35S_R	ATCTTTGCATTTTTCCTCATGTTGGG
c660	qPCR_660_35S_F	CTTGGCAAACTGTGGATGTTAAG
	qPCR_660_35S_R	CTCGTAAGGAGGATAAACCTGATATT
c692	qPCR_692_35S_F	GCAAGTCTGACCCTGAAAGA
	qPCR_692_35S_R	GGCACAACAAGTGAAAGGATAC
c908	qPCR_908_35S_F	CTGATATCTCTTCTCTGAAGTCCA
	qPCR_908_35S_R	GTTATTCTCTCTGGTGATAAAGACCT
c1459	qPCR_1459_35S_F	GAAGAGGAGTTCTCATGGTAAG
	qPCR_1459_35S_R	GAAGACGGATTGGCATTTTGATCC
EF1	qpcr_EF1_F	GACAAGCGTGTTATTGAGAGG
	qPCR_EF1_R	CACAGTGCAGTAGTACTTAGTG
L23	qPCR_L23_F	AAGGATGCCGTGAAGATGT
	qPCR_L23_R	GCATCGTAGTGGAGTCAAC
Sec3	qPCR_Sec3_F	GCTTGCACACGCCATATCAAT
	qPCR_Sec3_R	TGGATTTTACCACCTTCCGCA
PVY_uni	qPCR_PVY_F	CATAGGAGAACCTGAGATGCCAACT
	qPCR_PVY_R	TGGCGAGGTTCCATTTTCA
EDS1	RT_PCR_EDS1_F	CGATGAATTTAGCCCTGAATGACTTAGG
	RT_PCR_EDS1_R	TCACACTTGGTCTGGTACTCCTGTA
NRG1	RT_PCR_NRG1_F	GTTCTCTGCTCAATCTTAAGAGAATCAG
	RT_PCR_NRG1_R	GATCACATGTTTCAGCTGGTACTTCC
